# Adaptive Spike Threshold Enables Robust and Temporally Precise Neuronal Encoding

**DOI:** 10.1371/journal.pcbi.1004984

**Published:** 2016-06-15

**Authors:** Chao Huang, Andrey Resnik, Tansu Celikel, Bernhard Englitz

**Affiliations:** 1 Department of Neurophysiology, Donders Institute for Brain, Cognition and Behaviour, Radboud University, Nijmegen, the Netherlands; 2 Laboratory of Neural Circuits and Plasticity, University of Southern California, Los Angeles, California, United States of America; University of Tübingen and Max Planck Institute for Biologial Cybernetics, GERMANY

## Abstract

Neural processing rests on the intracellular transformation of information as synaptic inputs are translated into action potentials. This transformation is governed by the spike threshold, which depends on the history of the membrane potential on many temporal scales. While the adaptation of the threshold after spiking activity has been addressed before both theoretically and experimentally, it has only recently been demonstrated that the subthreshold membrane state also influences the effective spike threshold. The consequences for neural computation are not well understood yet. We address this question here using neural simulations and whole cell intracellular recordings in combination with information theoretic analysis. We show that an adaptive spike threshold leads to better stimulus discrimination for tight input correlations than would be achieved otherwise, independent from whether the stimulus is encoded in the rate or pattern of action potentials. The time scales of input selectivity are jointly governed by membrane and threshold dynamics. Encoding information using adaptive thresholds further ensures robust information transmission across cortical states i.e. decoding from different states is less state dependent in the adaptive threshold case, if the decoding is performed in reference to the timing of the population response. Results from *in vitro* neural recordings were consistent with simulations from adaptive threshold neurons. In summary, the adaptive spike threshold reduces information loss during intracellular information transfer, improves stimulus discriminability and ensures robust decoding across membrane states in a regime of highly correlated inputs, similar to those seen in sensory nuclei during the encoding of sensory information.

## Author Summary

A neuron is a tiny computer that transforms electrical inputs into electrical outputs. While neurons have been investigated and modeled for many decades, some aspects remain elusive. Recently, it was demonstrated that the membrane (voltage) state of a neuron determines its threshold to spiking. In the present study we asked, what are the consequences of this dependence for the computation the neuron performs. We find that this so called adaptive threshold allows neurons to be more focused on inputs which arrive close in time with other inputs. Also, it allows neurons to represent their information more robustly, such that a readout of their activity is less influenced by the state the brain is in. The present use of information theory provides a solid foundation for these results. We obtained the results primarily in detailed simulations, but performed neural recordings to verify these properties in real neurons. In summary, an adaptive spiking threshold allows neurons to specifically compute robustly with a focus on tight temporal correlations in their input.

## Introduction

The essential computation performed by neurons is the (non-linear) integration of synaptic inputs and subsequent generation of a spike. This summation is determined by multiple factors, including the passive membrane properties, active currents, and the neuronal geometry [1–3]. The currents at the axon hillock have voltage-dependent activation dynamics, which accelerate and then self-sustain the depolarization, eventually leading to a spike. The voltage at which this acceleration occurs is commonly referred to as the voltage threshold of spiking or spike threshold. Recently, several studies have empirically demonstrated that this threshold is not constant as recorded in cortical [4–8] and in subcortical [2,9,10] neurons. Rather, the spike threshold exhibits a dependence on the slope of the depolarization, such that fast depolarizations produce spikes at lower thresholds, and slower depolarizations at higher thresholds. The relationship is monotonically decreasing over a range of a few millivolts. This phenomenon is qualitatively different from the thoroughly studied spike-frequency adaptation, which acts on longer time scales [11–13] and depends on suprathreshold activity. The effects of the adaptation investigated here, will manifest already before the ‘first’ spike, e.g. at response onset.

Recently, Fontaine and colleagues [2] have demonstrated that this threshold dependence can be captured using a threshold which itself ‘chases’ the membrane potential, however, does not instantly adapt to the new membrane potential but approaches its new value with a certain time-constant. Consequently, if one considers two fluctuations of equal size, but different speed, the faster fluctuation will encounter a lower threshold (still in the approach of its final value) and thus already lead to a spike, whereas the slower fluctuation may encounter a higher threshold and thus lead to a spike at a greater depolarization (or fail to spike). Hence, the propensity to spike depends on the recent history of the membrane potential and in particular on the most recent rate of depolarization leading up to a potential spike. Multiple mechanisms could underlie this modulation in spike threshold, including adaptive changes in sodium channel inactivation [5–7,14] and potassium conductances [4,15]. Given that the existence of these conductances is common among spiking neurons, an adaptive spike threshold is likely to be ubiquitous features of neurons.

An adaptive threshold could have important computational consequences, since this would rapidly modulate the steepness of the input-output function of neurons [14]. Several authors [2,5–7,15] have speculated on the possible relevance of an adaptive threshold in the sense described above, however, to our knowledge, no study has thoroughly investigated its computational consequences.

Hence, here we set out to evaluate the effect of an adaptive threshold on the level of single neurons in feedforward excitatory networks in the context of stimulus processing. We investigate how a single neuron’s response to different rates and patterns of inputs varies as the action potential generation is gated by fixed, i.e. constant, or adaptive thresholds. Furthermore, we investigate how the state of the neuron (resting membrane potential prior to stimulus onset) influences the response behavior using both recordings from barrel cortex as well as neuronal simulations. The results suggest that neurons with adaptive threshold perform better than with a constant threshold when the stimulus is encoded by highly correlated inputs. Such a neuron encodes the stimulus more robustly, even in the context of noisy fluctuations of the membrane potential. For the state differences, we find the temporal reference used for decoding—internal or external—to play a major role: If the time reference is computed internally based on the timing information from the correlated spiking of local populations, an adaptive threshold turns out to be beneficial. Together these results show the usefulness of adaptive thresholds in performing temporally precise and robust computations.

## Results

We performed a combination of biological and simulation experiments to quantify the ability of neurons to encode stimulus information using information theoretic methods. Pyramidal neurons were recorded in L2/3 of barrel cortex *in vitro* while stimulating in layer 4 of the same column to experimentally. Further, we simulated different neuronal models with adaptive (as observed in the recordings) or fixed spiking threshold. For both biological and simulated neurons, we test the quality of the stimulus encoding for different coding schemes of the input and its robustness across membrane states. Neural integration turns a distributed current into a membrane potential and finally into a sequence of spikes. Below, we address the relation between the information in the input population and the output spike-train, thus leaving out the intermediate current level.

### Input-output behavior in different parametrizations

**Fig 1:**
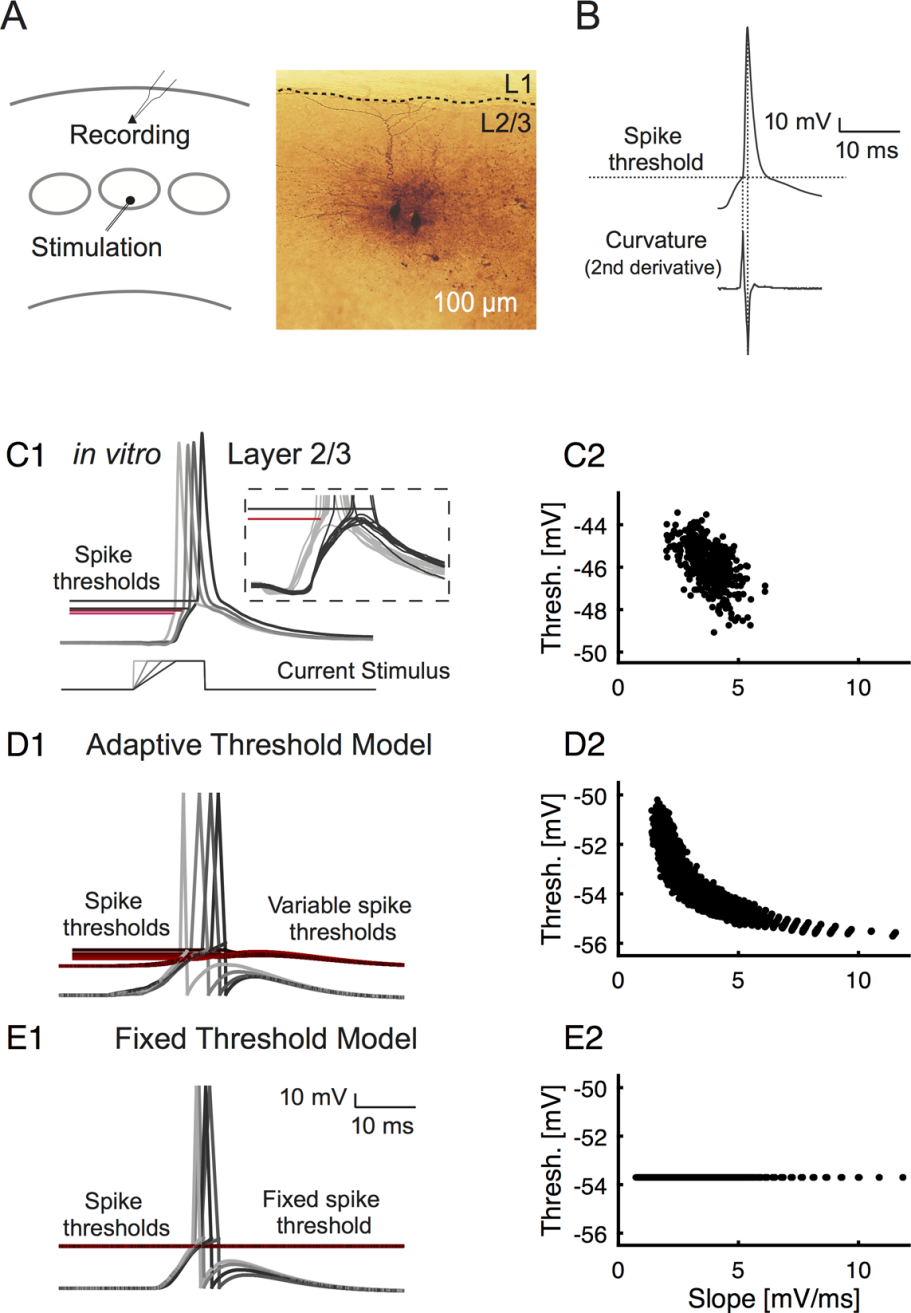
The spike threshold depends on the history of the membrane potential in both real and simulated data. **(A)** We performed patch-clamp recordings in layer 2/3 pyramidal neurons in vitro, in response to population input from stimulation in layer 4 (left). The pyramidal neuron identity was confirmed in a subset by filling the targeted neuron using biotin (middle). **(B)** From recorded action potentials (top), the spike threshold is determined as the maximal positive peak of the second derivative of the membrane potential (bottom). **(C1)** A cortical neuron stimulated with current inputs of different slopes (bottom, different shades of gray) lead to action potentials (top, corresponding grays) with different thresholds for spike initiation (top, red lines in corresponding brightness to grays of voltage traces, inset shows zoom in of spike initiation). The response is delayed w.r.t. to the stimulation due to the propagation delay from L4 to L2/3. The inset shows a magnified view of the threshold region. (**C2**) As in previous studies, thresholds were found to vary with the slope of the preceding membrane voltage. In the current stimulation settings, only a limited range of input slopes was realized. **(D1)** Neurons with an adaptive threshold were simulated on the basis of the model by Fontaine et al. [2], after adapting the parameterization to cortical excitatory neurons (see Methods). In addition to the voltage traces (grays), the adapting thresholds are also shown (reds, brightness corresponding to the gray traces). (**D2**) Applying the same analysis as in the in vitro data to measure the threshold, indicates that designed and measured threshold agree. The relationship between EPSP slope and spike threshold is overall captured by an exponential function especially when the wider range of EPSP slopes was used, which could be explored in the model (compare C2 and D2), see also [5]. **(E1)** Neurons with a fixed threshold were also simulated. The threshold was set to equalize firing probability with the adaptive threshold model. (**E2**) Re-estimating the threshold, we obtain the expected constant threshold.

While the adaptiveness in threshold is typically described in relation to the slope of the membrane potential [5–7,9] (Fig 1), for computational purposes it is more insightful to describe it in relation to the properties of the synaptic input to a single compartment postsynaptic neuron. On a mechanistic level, Fontaine et al [2] identified the membrane potential itself as a valuable predictor of the threshold, which then also defines the adaptation of the threshold in the neural model (see Materials and Methods). Subthreshold dynamics are expected to differ slightly between adaptive and fixed threshold, as the threshold dynamics (Methods, Eq 1) are already active below threshold.

While this choice is practically useful, it does not—or only indirectly—allow one to address questions such as the dependence of the spike threshold on the input rate, pattern, or correlation. Therefore, we first substitute the amplitude and slope of the incoming EPSP, by the number of synaptic/neuronal inputs N_inputs_ and their temporal dispersion σ, which parametrizes the Gaussian distribution from which the EPSP arrival times are drawn.

**Fig 2:**
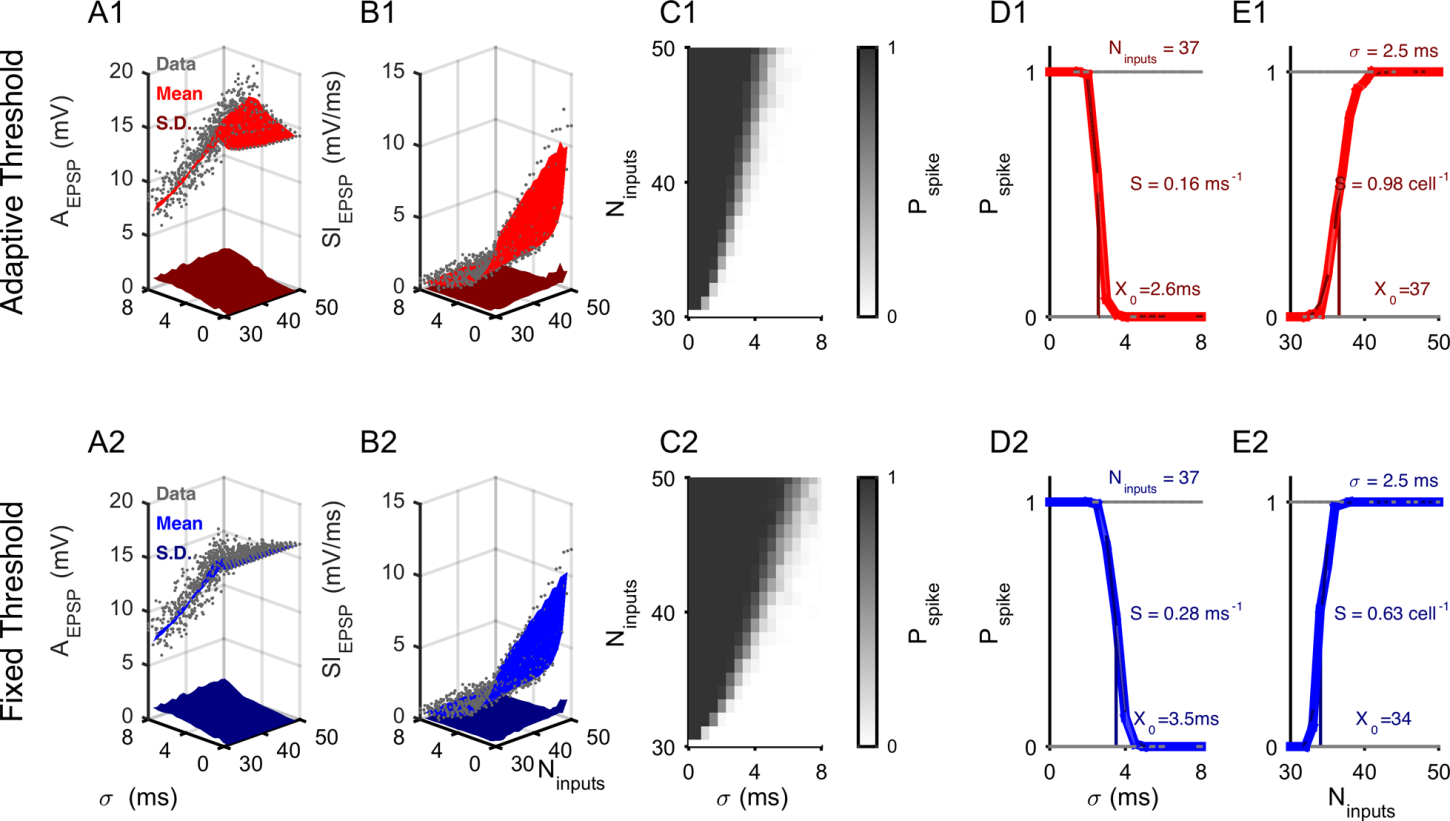
The input-output relationship sharpens due to the adaptive threshold. In previous studies, the dependence of spike threshold on previous membrane potential dynamics was most frequently linked to EPSP amplitude (A_EPSP_) or slope (Sl_EPSP_). Both measures can be reliably predicted by the combination of temporal input spread (σ) and the number of contributing neurons (N_inputs_) in point neurons. **(A)** The A_EPSP_ (data: gray, mean: red) is predicted with small standard deviation (S.D.) (maroon) by σ and N_inputs_ across a wide range of values for the adaptive threshold model (**A1**). Prediction quality was similarly good for the fixed threshold model (mean: blue, S.D.: dark blue, **A2**). **(B)** Similarly, the Sl_EPSP_ amplitude is predicted well with small S.D. on the basis of σ and N_inputs_, for both adaptive (**B1**) and fixed (**B2**). **(C)** Spike probability follows generally a similar shape as a function of σ and N_inputs_. However, the speed of transition between spiking and non-spiking domain is overall greater for the adaptive threshold model (**C1** vs.**C2**), translating into a steeper decision criterion as a function of the input parameters. **(D)** Spike probability as a function of only σ, is well fitted by a sigmoid, with the adaptive model (**D1** vs. **D2**, for N_input_ = 37) exhibiting a steeper slope as a function of different σ’s (adaptive: s = 0.18, fixed: s = 0.28) as well as a lower midpoint, indicating overall operation on a faster time-scale However, the inverse is the case for the dependence on N_inputs_ (**E1** vs. **E2**, for σ = 2.5 ms). Error bars represent 2x SEMs and are barely visible, only 1/50 of points plotted in A/B to improve display.

The parameterization of the input by N_inputs_ and σ is valid, since these two parameters predict the amplitude (Fig 2A1 and 2B1) and slope (Fig 2A2 and 2B2) of the resulting EPSPs with little variance (dark surface in the bottom of each panel). The quality of the prediction and the shape of the dependence are largely independent of whether the adaptive or the fixed threshold model is considered (Fig 2A1 vs. 2B1 and 2A2 vs. 2B2). Subthreshold dynamics are expected to differ slightly between adaptive and fixed threshold, as the threshold dynamics already act on the membrane equation (Eq 1) below threshold. This is in contrast to threshold models in which the spike threshold is not part of the membrane equation (e.g. leaky integrate and fire neuron), where the adaptation of spike threshold does not influence the subthreshold dynamics. As expected, increases in N_input_ and decreases in σ increase both the amplitude (A_EPSP_) and the slope (Sl_EPSP_) of the EPSP. Hence, the values of N_inputs_ and σ map approximately bijectively (one-to-one) to corresponding values of A_EPSP_ and Sl_EPSP_ and thus span the space of EPSPs w.r.t. to these two experimentally relevant parameters.

Based on these parameters, we investigated the transition between sub- and suprathreshold activity via the spike probability. Throughout this study the only source of variability in the simulations is trial-to-trial variation in synaptic strength (including failures, see Methods), with the exception of an investigation of noise susceptibility (see below), where Poisson spikes are added to the presynaptic input.

### Adaptive threshold provides sharper decision function in time but not in rate

The adaptive threshold model exhibits overall a steeper transition to spiking as a joint function of σ and N_inputs_ than the fixed threshold model (Fig 2C1 vs 2C2). Analyzing the two parameters separately, however, shows that the transition is only steeper for the temporal precision of the input, σ (Fig 2D1 vs 2D2), but not for the input rate, N_inputs_ (Fig 2E1 vs 2E2). The dependence of spike probability on σ and N_inputs_ exhibited similar shapes: Spike probability as a function of only σ, is well fitted by a sigmoid, with the adaptive model exhibiting a steeper slope as a function of different σ’s (adaptive: s = 0.16, fixed: s = 0.28). Fitting sigmoids to the spike probability as a function of the N_inputs_ shows an inverse relationship in steepness between the adaptive (s = 0.98) and the fixed (s = 0.63, E1 vs. E2) threshold neuron. In addition, the midpoint of the transition is located at a lower σ for the adaptive than for the fixed threshold (2.6 vs. 3.5 ms), while the midpoints for the dependence on N_inputs_ differ only slightly (34 vs. 37 inputs). The latter is a consequence of setting the fixed threshold matched to the average of the adaptive threshold.

The adaptive threshold model is thus able to respond to a wider dynamic range of the number of inputs contributing to a stimulus, restricted to a smaller range of integration time-windows (given here by the lower lowpass limit w.r.t. σ, compare Fig 2D1 to 2D2).

### Information transmission for rate and pattern encoding

Neural integration projects a high-dimensional input onto a lesser dimensional membrane potential and eventually onto a binary time series as spikes. While a certain amount of information will always be ignored in this process, the goal of mutual information analysis is to quantify the ability of neurons to give distinguishable responses to different inputs. In the following, we will focus on two encoding schemes on the input side, population rate and population pattern (which will be abbreviated as rate and pattern below).

**Fig 3:**
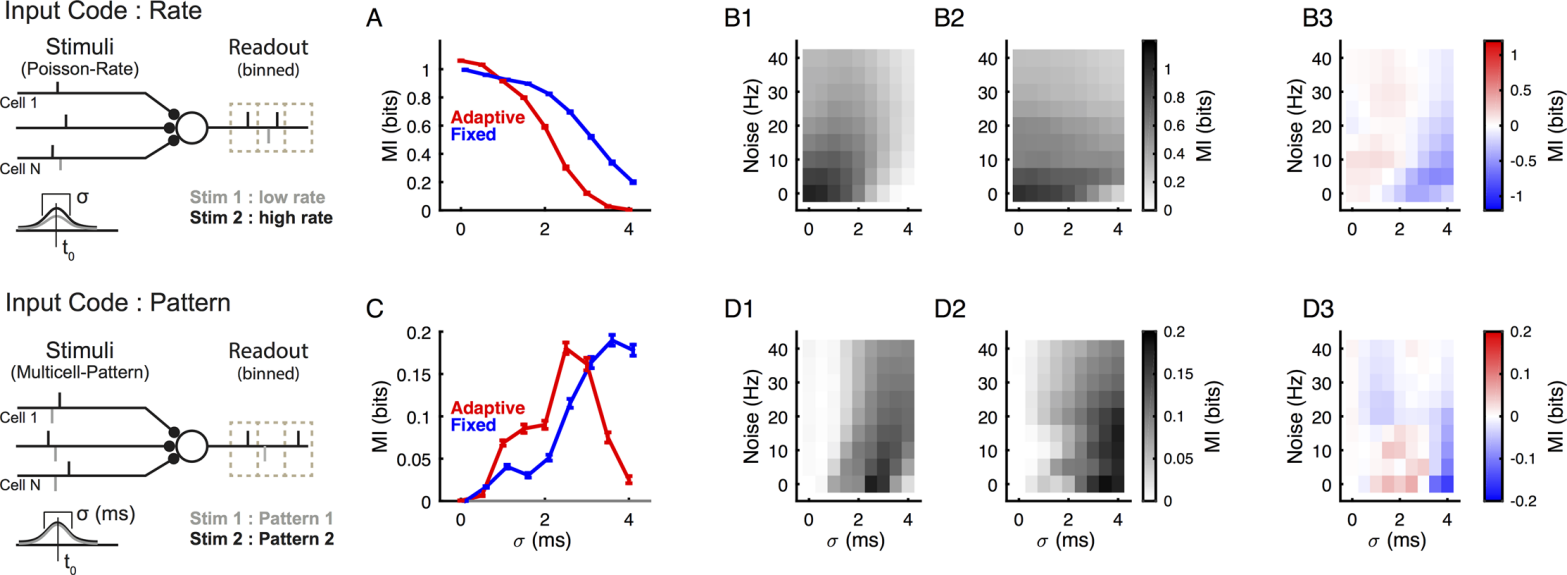
The adaptive threshold neuron is more informative for high temporal precision and low noise than the fixed threshold neuron. Two types of encoding (left) were investigated on the input side, either a classical rate encoding (top), where the number of input spikes carry the information but spike times are drawn randomly from a normal distribution centered at t_0_, and a pattern encoding (bottom), where the spatiotemporal pattern of inputs encodes the information and spike times are hence precise across repetitions. In both cases, the temporal precision (σ) according to which the stimuli are drawn is important (bottom). **(A)** Responses of the adaptive threshold model neurons (red) encode information mostly at low temporal spread σ, while the fixed threshold neurons (blue) possess a wider range of encoding w.r.t. to σ if information is rate-encoded. **(B)** This relationship holds across a wide range of noise inputs, with adaptive threshold neurons encoding generally better for lower values of σ, and fixed threshold neurons for higher (**B3,** the differences in mutual information between adaptive and fixed threshold models). Color mapping here represents information. Noise was modeled as independent Poisson spike trains with constant rate for each input neuron; there were 100 input neurons in total. **(C)** A similar relationship in information encoding between the models is observed when information is decoded from the temporal pattern of incoming spikes. Again the adaptive threshold model performs better for low σ. **(D)** This relationship holds only for a limited range of noise, after which point the differences between the models becomes fairly small.

In the case of population rate encoding (Fig 3 top), the studied neuron receives input from a population of presynaptic neurons, and inputs differ in their overall firing rate. Although each presynaptic neuron generates at most one spike in a given trial, the input rate is varied by changing the number of neurons that contribute to the current input. The spikes from the presynaptic neuron population are normally distributed as a function of time around a common average time t_0_ with standard deviation σ. Spike times are drawn randomly for each trial, while only the number of participating neurons stays the same across trials for a given input. Under this encoding schema, the only difference among stimuli is the population firing rate of the input neurons, and the temporal patterns of presynaptic spikes provide no information. For the simulations reported, in total 11 different stimuli were given (range of active neuron number, 40–60, incremented by 2), thus the total stimulus entropy was log_2_(11)≈3.46 bit.

Adaptive threshold neurons performed slightly better for low σ, but substantially worse for larger σ for rate encoded inputs (Fig 3A). In these simulations noise exists as variability in the synaptic weights (modeled as described in Feldmeyer et al. 2002), but no additional inputs were added. Adding additional independent Poisson noise inputs generally reduces the recoverable mutual information (Fig 3B1 and 3B2), but does not change the qualitative finding of a better decoding of rate by fixed threshold neurons for larger σ (Fig 3B3). Overall, both adaptive and fixed threshold neurons exhibit a low pass-behavior in σ, which results from the fact that a greater σ at some point exhausts the integration window of each model.

In the case of the population pattern encoding (Fig 3 bottom), the different inputs are created by setting distinct predetermined patterns across neurons, which all have a fixed population firing rate (N_input_ = 50). Under this condition, the same number, but a different subset of neurons is activated by different stimuli. Across trials, each presynaptic neuron gives the same spike time for a given stimulus. Hence, distinction between different stimuli has to rely on the temporal differences between different input spike patterns. As above, 11 different stimuli were delivered, resulting in ~3.46 bit stimulus entropy.

Adaptive threshold neurons perform substantially better for low and worse for greater σ. Adding independent Poisson noise changes the relation between the models qualitatively, with the clear difference between them progressively giving way to a weak, but inverse relationship (Fig 3D). This is due to the dependence of the MI peak on the noise strength: in the adaptive threshold model larger noise shifts the peak more quickly to greater σ's than in the case of the fixed model (Fig 3D1 vs. 3D2). Both model neurons exhibit a band-pass behavior w.r.t. to σ, in contrast to the low-pass behavior for rate encoding, which follows from the fact that patterns with very small σ effectively get squeezed to the same, single-time bin pattern.

In summary, adaptive threshold neurons are useful encoders for precisely timed, or well correlated, inputs. Interpreted inversely, the shortened integration window of the adaptive threshold model, allows it to ignore inputs outside a certain period, and thus make decisions more rapidly (see also [16]). On the other hand, a constant threshold would be advantageous if the processing has to happen over a wider range of temporal correlations in the input. We have performed a limited set of simulations of Hodgkin-Huxley neurons with a range of parameters, which transitions its behavior from an adaptive to a fixed threshold (similar to [17]). Overall, the Hodgkin-Huxley model performed quite similar to the simpler model described above (S1 Fig).

### Membrane and threshold dynamics influence temporal selectivity

The adaptive threshold is governed by a few parameters (see Eqs 2 and 3 in Methods), which determine its dynamics and asymptotic value. Next, we address the interplay of the temporal dynamics of the threshold, given by the time constant *τ*_*θ*_ (Eq 2), with the passive integration of the neuron, given by the membrane time constant *τ*_*m*_ (Eq 1). For this purpose, we repeated the simulations above for a matrix of *τ*_*θ*_ and *τ*_*m*_ values, and evaluated the effect on firing rate, represented information and most informative temporal dispersion σ.

**Fig 4:**
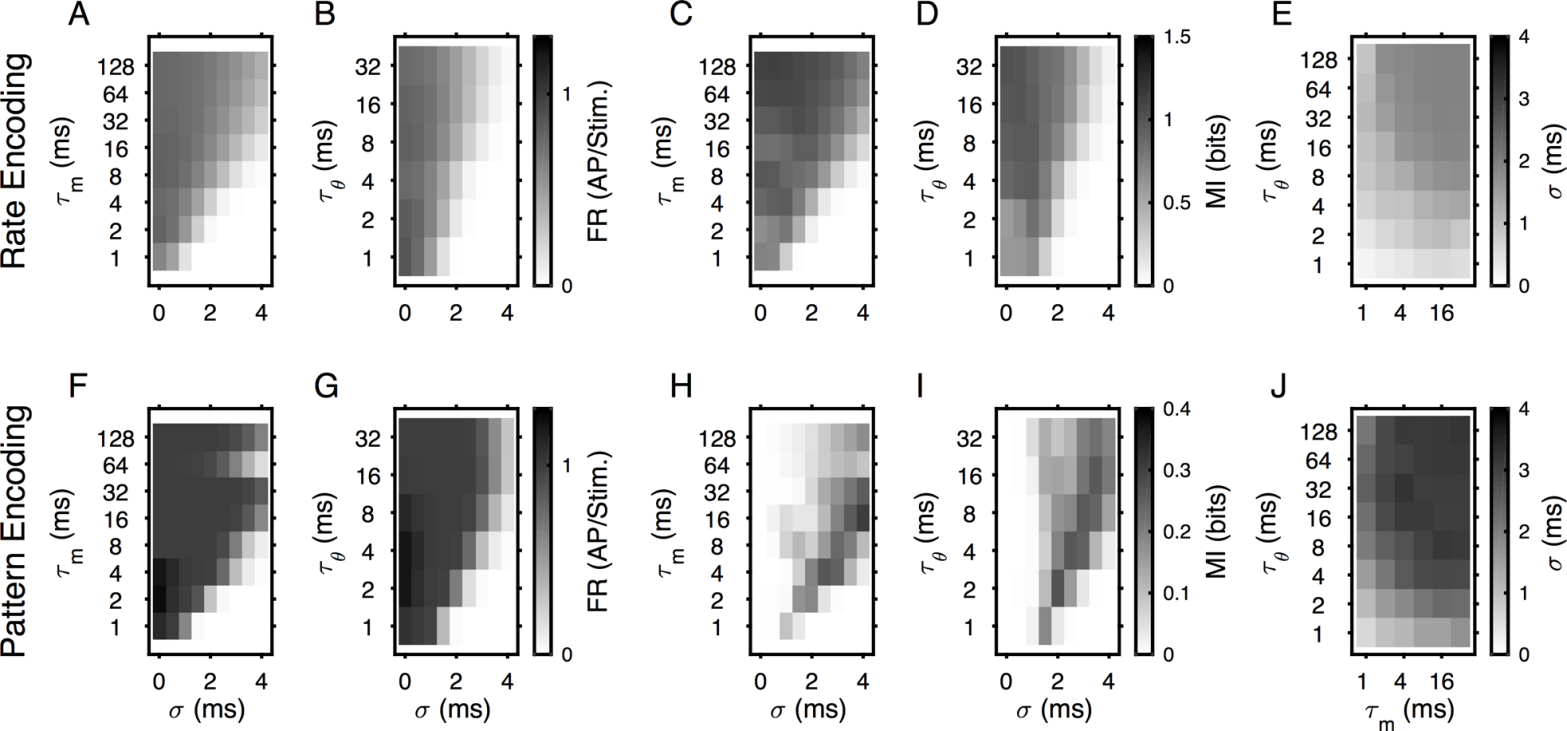
Membrane and threshold time constants influence temporal selectivity similarly. **(A-B)** For rate encoding, the firing rate of the model neurons was limited by τ_m_ (A) and τ_θ_ (B), if either of them were too small. Short τ_m_ prevent integration, while short τ_θ_ lead to a threshold that quickly follows the input. **(C-D)** The limitation in firing rate by τ_m_ (C) and τ_θ_ (D) translates to a limitation of represented stimulus information. Only for short values of τ_m_ and τ_θ_ the lowpass shape gives way for a bandpass shape, which is a consequence of the decoding bin size chosen here (2ms): for small σ and low τ_m/_τ_θ_ the responses to different stimuli will tend to fall in a small number of bins. **(E)** We extract the average σ that maximized MI for a combination of τ_m_ and τ_θ_. The resulting dependence is monotonically in τ_m_ and τ_θ_, consistent with a change in the σ edge of the lowpass relationship (Fig 3A). **(F-G)** For pattern encoding, the firing rate is limited analogously by τ_m_ (F) and τ_θ_ (G). **(H-I)** The value of σ that leads to highest MI shifts with both τ_m_ (H) and τ_θ_ (I), indicating a match between the membrane dynamics and the temporal scale of input patterns that can effectively be encoded. Larger σ values generate temporal patterns that are more distinguishable but the spikes are more dispersed in time,and both τ_m_ and τ_θ_ set the temporal limit in which the spikes can be integrated. **(J)** The best MI is achieved at different average σ across the range of τ_m_ and τ_θ_, monotonically increasing for both parameters.

For rate encoded stimuli, the average firing rate was limited similarly by both *τ*_*θ*_ and *τ*_*m*_ (Fig 4A and 4B, in plots A-D and F-I the other time constant is set to 4 ms). The reason differs slightly between them: while small *τ*_*m*_ prevent integration for larger σ, small *τ*_*θ*_ allow the threshold to change rapidly, adapting fully to the input and thus preventing the generation of a spike. The represented information was limited by the firing rate and thus affected similarly by both time constants (Fig 4C and 4D). Overall, MI therefore follows a lowpass behavior w.r.t. σ, although certain combinations of time constants with σ led to transient bandpass characteristics (e.g. for *τ*_*θ*_ = 2 ms, where the bin size of the MI analysis cannot distinguish the resultant spike times any more). The joint influence of *τ*_*θ*_ and *τ*_*m*_ on temporal integration exhibited a monotonic increase of σ_cm_ (Fig 4E), which was defined as the center of mass in MI w.r.t. σ for each choice of *τ*_*θ*_ and *τ*_*m*_, computed as the average over σ, weighted by the normalized MI.

For pattern encoded stimuli, the dependence of firing rate was analogous to the rate encoding case, exhibiting a low-pass limited by both time constants (Fig 4F and 4G). The σ of peak MI increased in correlation with the time constants, broadening alongside (Fig 4H and 4I). The joint dependence of σ_cm_ on *τ*_*θ*_ and *τ*_*m*_ exhibited a similar dually monotonic increase (Fig 4J), as predictable from the individual dependencies.

Overall, the best time scale for integrating input varies smoothly and similarly with the governing time constants *τ*_*θ*_ and *τ*_*m*_. Further, this relation is independent of the input encoding scheme. We also investigated the influence of the input population's size (see S2 Fig). Large input populations were favorable for rate encoding; small ones for pattern encoding, however, adaptiveness of the threshold did not have an effect on the N_input_ dependence.

### State dependence of information transmission

Cortical populations undergo membrane state changes, depending on the wakefulness or attentional context of the brain [18,19]. The state changes manifest themselves on the single cell level as a shift in the subthreshold membrane potential, thus bringing each cell to a different voltage distance to its spiking threshold. While the sources of the state changes are not fully understood, their presence will either change network computation, or require network computation to be robust against the changes. An adaptive threshold could be a contributor to robust processing under different states. We therefore investigated the robustness of adaptive compared with fixed threshold neurons for different initial membrane voltages for rate-encoded inputs (N_input_ range 50–60, with increment step size of 2; in total 6 stimuli with log_2_(6) ≈ 2.58 bit entropy). An ideal observer could decode the stimulus from the spiking activity of the simulated neuron, with or without knowing the resting membrane potential state of the simulated neuron. We defined a (relative) robustness index *RI* (see Methods) to quantify the neuron’s robustness to state changes, which was defined as the ratio between *I(S;R)* and *I(S; R*, *state)*. *I(S;R)* is the stimulus information, which can be gathered from the neural response without knowing the state, i.e. the information robustly present in the neural response independent of state knowledge, and *I(S;R*,*state)*, the stimulus information that can be gathered when also knowing the state. A neuron that encodes perfectly robust to state-differences would not show any improvement by knowing the state, and thus have an *RI* close to 1. The values of *RI* typically fall in [0, 1] (in some cases the maximal value is exceeded due to numerical imperfections), since adding state-information increases the mutual information due to part of the variability being explained by state knowledge (pointed out for example by [20]).

**Fig 5:**
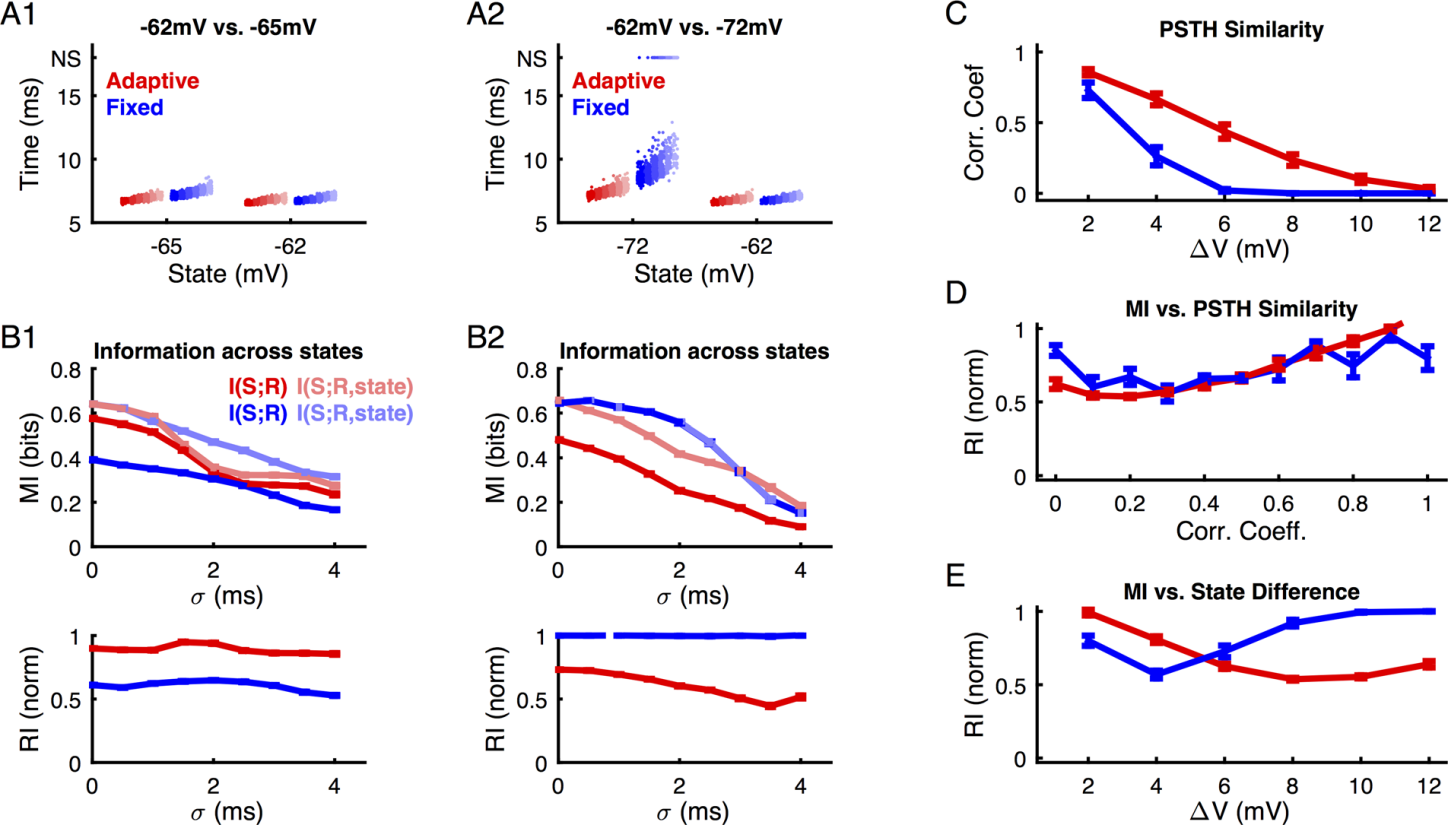
Information decoding across different membrane states and spike threshold types for a ‘stimulus-centered’ time reference. **(A)** For small differences in state (**A1**: -62mV vs. -65mV) both adaptive (red) and fixed (blue) threshold models show a shallow dependence on stimulus strength (different rates, range [50:2:60] inputs, total stimulus entropy ~ 2.58 bit, dark color = 60, light color = 50 EPSPs). The adaptive threshold model compensates partly for the difference in initial voltage, and thus exhibits smaller differences in spiking behavior across states. During larger fluctuations in membrane potential (**A2**: -62mV vs. -72mV) this behavior is qualitatively retained. The different states also scale the slope (average spike times vs. input strength) and spike time variability for each model, with the fixed model being influenced more strongly in both cases. NS, non-spiking trials. **(B)** Mutual information is estimated across all states with the state known (light colors) or unknown (dark colors) to the decoder. For small state differences (**B1**) the adaptive model’s MI is independent of state knowledge (RI close to 1, bottom), while the fixed model shows a strong dependence to membrane state (RI around 0.6, indicating ~40% of MI added by state knowledge). For larger state differences, this relationship inverts (**B2**). Note that in **B2** upper panel the dark blue curve almost fully overlaps with the light blue curve. **(C)** To understand this inversion, we relate the response distributions to state difference. The correlation coefficient between response PSTHs measures similarity across state differences. The adaptive threshold neuron (red) retains similar responses for larger state differences than the fixed threshold neuron (blue). **(D)** The correlation coefficient of response distributions predicts qualitatively the RI values, which indicates the advantage of knowing state during decoding. **(E)** The adaptive model encodes information in a state-independent manner for small state differences, while the fixed threshold model becomes more state-independent only for larger state differences. This switch is caused by a shift from overlapping to non-overlapping temporal decoding ranges, as indicated by the correlation coefficients of the PSTHs across different states (see Fig 5C, where a vanishing correlation indicates a lack of overlap between the responses across states). Hence, the adaptive threshold model compensates for a part of the initial state, however, does not encode more information independently if a stimulus-based, fixed-time decoding reference is used. Error bars indicate SEMs across all state differences of a given size.

The adaptive threshold model exhibited a reduced dependence of response latency as a function of different initial states (Fig 5A, the shown spikes are relative to presynaptic stimulus onset). For a small difference of only 3mV (-65 vs. -62mV, Fig 5A1) the spike times of the adaptive threshold neuron are almost identical across states (red, different shades indicate different N_input_, with more solid colors corresponding to greater N_input_), while the times are already noticeably shifted for the fixed threshold neuron (blue). Correspondingly the mutual information with state knowledge (Fig 5B1, solid colors), is only slightly larger than the mutual information without state knowledge (light colors) in the adaptive case, but substantially larger in the fixed case. The adaptive model has an *RI* close to 1, while the fixed model’s *RI* is much lower, which remains fairly constant as a function of σ (Fig 5B1 bottom).

If one considers larger differences in state (Fig 5A2, -72 vs. -62 mV), the changes in spike time due to the state changes become more severe. Especially for the fixed threshold model, the set of spike times becomes essentially disjoint, and in the more hyperpolarized state (-72 mV) the dispersion of the spike times increases substantially. In addition, failures of spike elicitation are observed (Data on top, marked as NS). For the adaptive threshold model the spike time shift becomes stronger than observed with smaller (3mV) state difference, but stays limited in comparison to the fixed model. Interestingly, from an information theoretic perspective, this leads to an inversion of the situation: The fixed model is now less dependent on the knowledge of state (Fig 5B2, top), again almost independent from σ (Fig 5B2, bottom). The basis of this inversion is that decoding across states can also work optimally, if the response time distributions are different, but disjoint across states.

The difference in robustness against variations in initial membrane potential of the models observed holds more generally across a wider range of state differences. The differential behavior described above rests on the overlap between the response distributions across different states (Fig 5C, quantified by the correlation coefficient across PSTH-bins CC_PSTH_). For small state differences the PSTHs are almost identical, leading to a correlation coefficient close to 1. For large state differences the PSTHs are disjoint and hence the correlation coefficients are close to 0. In between, the fixed threshold model exhibits a faster decay in correlation coefficient, thus indicating a faster drift of onset times.

Qualitatively, the correlation coefficient between PSTHs is a good predictor for the resulting robustness index (*RI*) during decoding (Fig 5D). High *RI*, i.e. high robustness, is achieved both for highly similar (CC_PSTH_ = 1) and dissimilar responses (CC_PSTH_ = 0). Correspondingly, *RI* varies non-monotonically as a function of state difference, with the adaptive threshold neuron being more robust for small state differences, and the fixed threshold neuron being more robust for larger state differences (Fig 5E).

Hence, an adaptive threshold improves the robustness to state differences in comparison to a fixed threshold only for a limited range of small state differences. For larger state differences, the confusion generated by small shifts in timing is outweighed by the decoding possible across different time-windows. This occurs in the fixed threshold case, where response times shift far enough across states to make them nearly disjoint, thus in principle providing it with an advantage in information transmission.

### Decoding across states with a moving reference frame

Classically, information theoretic decoding is performed in reference to the timing of the stimulus, e.g. spike-times are computed in relation to the time of stimulus onset (‘stimulus-centered’, [21–23]). While this provides an objective reference, the brain's internal decoding mechanisms may not have direct access to the (external) information about stimulus onset. Alternatively, responses can be decoded in relation to the response timing of other neurons [24,25]. Use of this 'response-centered' decoding is not only closer to the brain's internal perspective, but also provides more accurate decoding if the time of stimulus onset is uncertain [25].

**Fig 6:**
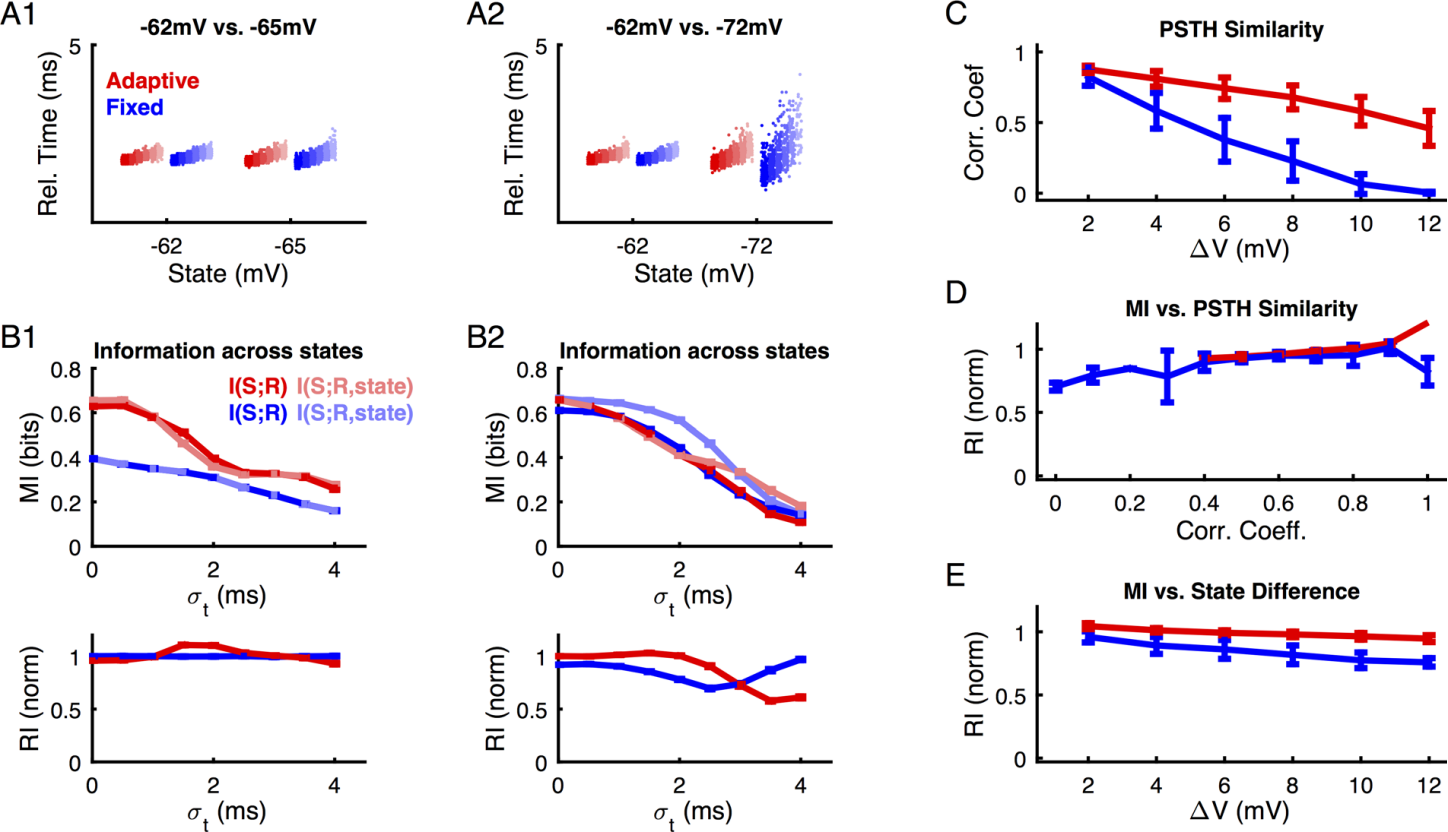
An adaptive threshold neuron represents information more robustly across membrane states for a ‘response-centered’ time reference. Since stimulus onset is not known internally, a population-based decoding reference has been suggested to serve a biologically relevant surrogate for stimulus timing [25]. With this reference, spike-timing is measured relative to the peak-time of the population response, i.e. local maxima of the population peristimulus time histogram (PSTH). **(A1)** For small differences in state, adaptive and fixed threshold models show little difference in their relative-time response and consequently the contribution of the knowledge about the state becomes negligible (**B1**). All colors are as in the preceding figure. (**A2**) For larger state differences, the response time distributions now differ significantly in their variance, with the fixed threshold model exhibiting a much larger increase in spread for the more hyperpolarized membrane state. Consequently, decoding across states becomes less robust for the fixed model than for the adaptive threshold model (**B2**). (**C**) While the ‘response-centered’ time reference increases the similarity of the PSTH across states for both the fixed and the adaptive threshold model, the latter profits more, widening the gap between the models for larger state differences. (**D**) As before, the correlation coefficient between the PSTHs remains a good predictor for the RI values. (**E**) In the case of the moving decoding reference, the adaptive model is generally more robust than the fixed threshold model across all state differences investigated, as indicated by a higher RI value. Error bars indicate SEMs across all state differences of a given size.

In the case of response-centered timing, the adaptive threshold model is found to be generally more robust to differences in state than the fixed threshold model. The internal reference time was defined as the peak-time of the population response (termed 'columnar synchronous response' in [25]). Since the neural population encompasses a range of tuning preferences, this leads to an average response time of the neural population to the stimulus set (see Materials and Methods). Effectively, the spike-times of the analyzed neuron are thus shifted to a common average response time of all simulated neurons, which will, however, depend on the membrane state and neural response properties, e.g. threshold type (see Fig 6A).

For small differences in state, both adaptive and fixed threshold models show little difference in their response time and variation in response time (Fig 6A1, red vs. blue, different shades indicate different stimuli, as in Fig 5 only rate encoding is considered here). Consequently, the *RI* is quite high (Fig 6B1, especially bottom). For larger state differences, the response time distributions differ strongly in their variance, with the fixed threshold model exhibiting a larger increase in spread for the more hyperpolarized state (Fig 6A2). The reduced variance across states and stimuli here exemplifies the effect of the adaptive threshold. The quality of unified decoding across states deteriorates for the fixed threshold model, thus leading to a larger information gain from the inclusion of state information, as indicated by the reduced *RI* values (Fig 6B2).

As a function of state-difference, both the fixed and the adaptive threshold model exhibited better conserved PSTHs across states (compare Fig 6C with Fig 5C) when ‘response-centered’ reference was used. However, the adaptive threshold model profits more and thus a wider gap between fixed and adaptive threshold model results in terms of PSTH correlation coefficients. As before, the correlation coefficient between the PSTHs remains a good predictor for the *RI* values (Fig 6D), although the shape slightly differs from the previous case (Fig 5D).

For 'response-centered' decoding, the robustness across states is generally higher for the adaptive than the fixed threshold model (Fig 6E), independent of the size of the state-difference (compare to Fig 5E). While the robustness partially depends on the particular combination of states (combinations averaged in Fig 6E), the variability is small compared to the effect size (errorbars in Fig 6E).

In summary, an adaptive threshold neuron can be decoded more robustly than a fixed threshold neuron, if the stimulus timing is known from the brain's internal perspective. Under these circumstances, the absolute timing is converted to a relative timing in the population, and the reduction in response variance in the adaptive threshold model renders responses across different states more comparable.

### State dependence of information transmission in cortical neurons

Cortical neurons have been shown to display spike threshold adaptation [4–8]. However, the effect of this adaptive threshold on the information transmission across resting membrane potential states has not been quantitatively investigated. We collected whole-cell patch clamp recordings from pyramidal neurons in L2/3 mouse barrel cortex in acute slice preparations, and delivered the stimuli after clamping the somatic membrane potential across different resting potentials. The stimuli consisted of currents with different rising slopes injected from a bipolar electrode placed in the L4 of the same barrel column as recorded cells, which were comparable to the “rate encoding” in the input population as mentioned earlier. In total 4 different stimuli were delivered, with ~2 bits of total entropy.

**Fig 7:**
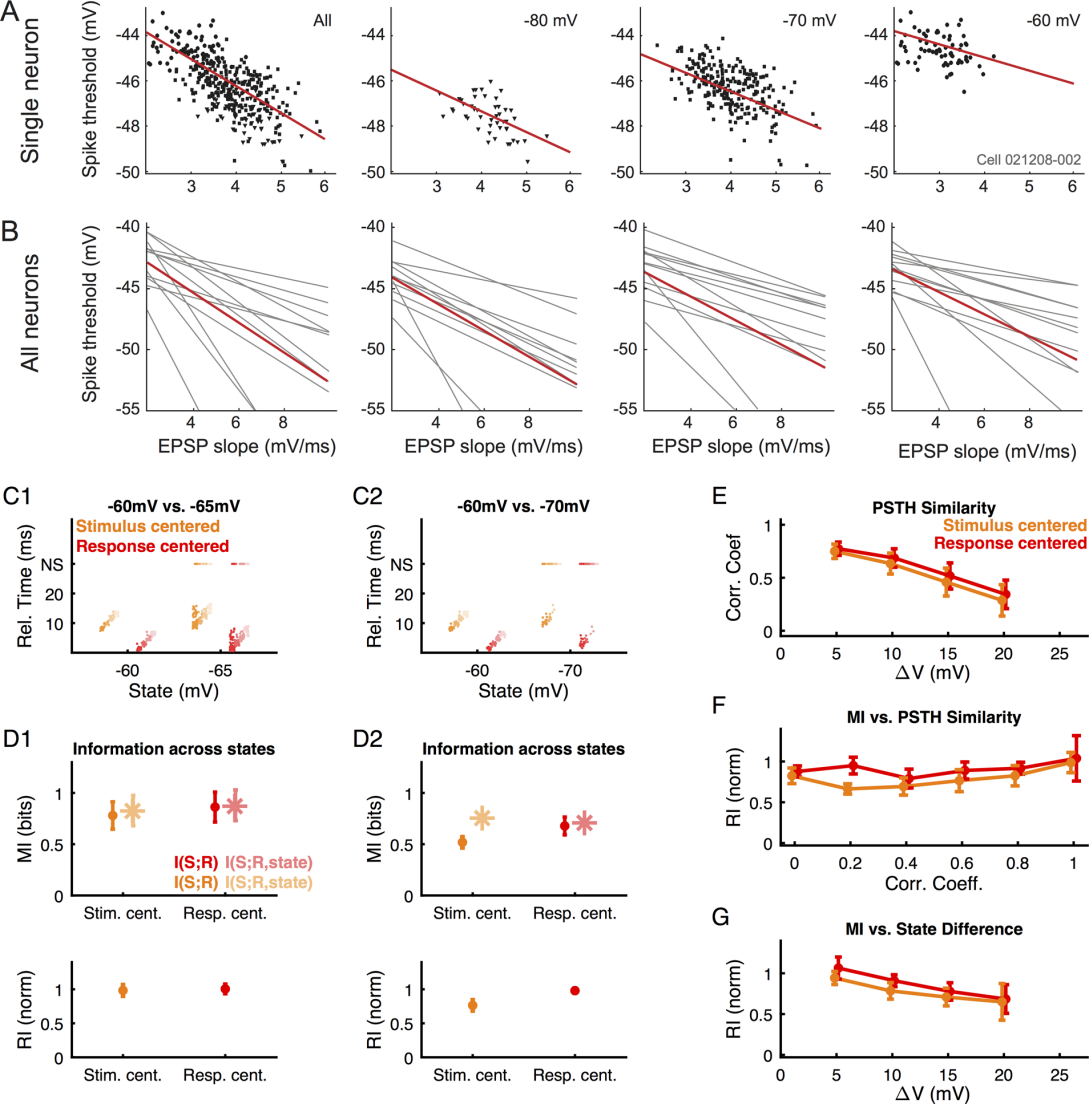
The state dependence of neural data corresponds closely to adaptive threshold behavior. Identical analyses were carried out as for the model data in the preceding figures. (**A**) Across three initial states of voltage (-80, -70, -60mV) a correlation between threshold and the EPSP slope was observed, i.e. a negative dependence between spike threshold and EPSP slope. Red lines, least-square linear fit to the data. (**B**) All recorded neurons (N = 11) exhibited this behavior. The average slopes across the different states were across the three states, i.e. -0.99 (0.27), -0.92 (0.19), and 0.90 (0.23) ms, respectively. Numbers in () are s.d. Red lines, average across all the neurons. (**C**) For small state differences, both the response patterns (**C1**), the decoded information (**D1** top) and the robustness (**D1** bottom, measured as robustness index RI) remain comparable across stimulus-centered (orange) and response-centered (red) decoding. N.S., non-spiking trials. (**D**) For larger state differences the advantage of decoding with an adaptive threshold becomes evident (**D2**). Stim. cent., stimulus-centered; Resp. cent., response-centered. (**E**) The similarity of PSTHs as a function of state difference reflects the behavior of the adaptive threshold model (Figs 5C and 6C, red) exhibiting a slow decay, based on a similar robustness in mean and variance of the spike-timing. (**F**) Robustness in decoding across states shows a similar dependence on the correlation coefficient as for the model data (compare orange to Fig 5D, and red to Fig 6D), validating the analysis across real and model data. (**G**) Robustness across states of the cortical neurons exhibits a shape closer to the adaptive threshold model, characterized by a slower and later decay (for static decoding especially) than for the fixed threshold model (Fig 5E).

Similar to previous studies, the recorded neurons exhibited an adaptive threshold that depended on the slope of the input for all states (-80, -70, -60mV; *r*^*2*^ = 0.60±0.18, 0.45±0.13, 0.52±0.10, respectively. Values are mean±s.d., n = 11) investigated (Fig 7A and 7B). Interestingly, this relationship appears not to be strongly dependent on the resting membrane potential, as the average slopes obtained from linear regression across the different states were comparable, i.e. -0.99 (0.27), -0.92 (0.19), and 0.90 (0.23) ms respectively (p = 0.48, one-way ANOVA with correlated samples, numbers in parentheses are s.d.).

The neurons also exhibited a high PSTH similarity, as well as a high robustness of information encoding across states, reminiscent of the adaptive threshold model. For small state differences, both the response patterns (Fig 7C1), the decoded information and the *RI* value remain comparable (Fig 7D1) across *stimulus-centered* (orange) and *response-centered* (red) decoding. For larger state differences the improved robustness of decoding to state variation with a *response-centered* threshold becomes evident (Fig 7D2).

The correlation coefficient between PSTHs at different states remained high for a wide range of state differences. This range was even wider than the one for the adaptive model for *response-centered* decoding (compare Fig 7E to Fig 6C). This difference between real and simulated neurons could either stem from a mismatch of the model parameters to the real neuron or an additional mechanism not accounted for by the model.

Overall, the relation between across-state robustness (as indicated by the *RI*) and correlation coefficient (Fig 7F) was similar for real and model data (compare orange to Fig 5D, and red to Fig 6D). Robustness across states exhibits a shape that stays closer to the adaptive than the fixed threshold model behavior. It rises slowly and decays much later, especially for the static decoding (Fig 7G compared to Fig 5E).

In summary, the information transmission behavior of cortical neurons exhibited similar features of robustness across states as the adaptive threshold model. An adaptive threshold could therefore be a mechanism which contributes to achieving this robustness, providing a functional reason for its widespread existence in the mammalian nervous system.

## Discussion

We investigated the computational properties of neuron models with an adaptive spike threshold. The results showed that a neuron with an adaptive threshold processes information specifically in well-correlated inputs both for rate and pattern encodings. Furthermore, the adaptive threshold provides a higher level of robustness than the fixed threshold model against variations in the initial state, if the decoding is performed relative to the average response-time. The experimental data behave similar to the adaptive threshold model with respect to robustness against changes in initial resting membrane potential state. Below we explore the consequences of these results for neural coding, and discuss a range of assumptions and limitations for the present study.

### Relation to previous studies

Threshold adaptation has been observed in a range of systems and modalities, and therefore appears to be the norm rather than the exception. In cortical neurons, the spike threshold is shown to be correlated with the average membrane potential prior to a spike [6,26], as well as inversely correlated with the rate of membrane potential depolarization in both excitatory [5–8] and fast-spiking interneurons [4]. The adaptive threshold increases the sensitivity to synchronized presynaptic inputs, while suppressing uncorrelated inputs, thus potentially increasing stimulus selectivity. Similar forms of spike threshold adaptation have been reported in the thalamus [9], the subthalamic nucleus [10] and the auditory brainstem [2]. Importantly, threshold adaptation has to be separated from spike frequency adaptation, which occurs as a consequence of spiking, but is not influenced by the subthreshold voltage. Further, the present study skips the synaptic current level, but works directly on the neural output, which is generated by the nonlinear spiking process.

Previous studies have speculated and partially linked the adaptive nature of the spike threshold to improvements in neural coding, but not performed corresponding analyses to quantitatively test this hypothesis. The clearest investigation of the mechanism is found in Fontaine et al. [2], where the difference between the adapting threshold and the membrane potential is identified as the 'effective signal', which determines under what conditions the neuron will spike. They demonstrate directly, in the context of neurons from the barn owl's inferior colliculus, that the response selectivity shifts towards temporally more tightly tuned coincidences. The present reparametrization of the EPSPs in terms of the input temporal precision is partially guided by this finding. Their analysis, however, does not proceed to a full decoding analysis as presented here. We recover their result in a more general form for the information analysis (Fig 3), with improved encoding for tightly tuned inputs in the adaptive threshold neurons than in the fixed threshold neurons, both with respect to rate and pattern encodings in the input.

Another interesting perspective is provided by the study of [Fontaine et al 16], which investigated the transition from encoding sound pressure to encoding the envelope of sound in the barn owl's cochlear nucleus. Remarkably, they find that this is enabled by the adaptive threshold of neurons in the nucleus angularis. Hence, the adaptive threshold can also take the role of transitioning between codes, rather than changing the precision within a code.

### Robustness in information transmission to initial state

While the functional significance of state changes is still debated, research over the last decade has indicated that (subthreshold voltage) state changes occur under a variety of conditions, ranging from different states of wakefulness [18], movement [27] or task involvement [19], to rapid changes during sensing [28]. While the activity of neurons will undoubtedly be modulated by the change in subthreshold membrane potential, it is not clear how the decoding will be affected. We presently addressed this question on the basis of modeled and real data, by computing the mutual information between stimulus and response for responses from neurons in different states. In particular, we investigated how insensitive the decoding was to the subthreshold state, by comparing decoding with and without state knowledge.

The results suggest that an adaptive threshold confers an increased robustness across states, especially if the decoding is performed based on an internal time reference. As we argue below, a decoding scheme consistent with the internal perspective of the nervous system has to rely solely on quantities which are internally available. Consequently, an adaptive threshold provides support to the idea that the processes of decoding can remain unchanged, even if the state changes. While other mechanisms (e.g. the hyperpolarization activated cation currents [29]) may also contribute to robust processing, an adaptive threshold may partially explain the relative constancy of perception across different states.

In a related study, Safaai et al [20] have investigated how much information about the stimulus can be gained by being aware of the current state. Their measure accounted for the state-dependent variability within the total stimulus information. The present robustness index RI constitutes 'the other side of the coin', in asking how consistently a cell responds across states, i.e. how much information is lost in not knowing the state.

### Internal vs. external timing reference

Neural communication occurs in time, and it is not clear if there is a common clock in the brain which could be used to determine the start and end of a message. The interpretation of a message depends on the temporal reference used, and can determine the meaning of the message, as well as the set of messages distinguishable, and hence the capacity of the channel. For example, a given spike pattern "1011" (binned over time) can be interpreted as "0101100" and "0001011" depending on the time reference. These messages may carry different information. This becomes particularly evident in the typical scenario of neural integration of messages (spike-trains) from multiple sources at the same time, where a time-shift between two spike-trains will affect their integration by the post synaptic neurons [30,31].

Since a neuron only has access to information from its own inputs (including modulatory inputs), a time reference has to be generated from the brain's internally available information. A time reference can be generated in multiple ways. For example, in vocal communication, pauses between words or sentences are used as markers to define starting points for interpreting parts of the entire message. Given the onset response properties of sensory neurons, these pauses will generate volleys of spikes, which can be used as a temporal reference. As suggested by Panzeri and Diamond [25], a similar interpretation could hold for the onset responses generated by tactile events in the somatosensory system [32,33] as cortical neurons integrate spatiotemporal information on behaviorally relevant time scales [34]. More generally, a given neuron could derive a time reference from the average timing of its synaptic input. The existence of such timing references is more generally suggested by the existence of large-scale oscillatory signals both on the cortical [35–37] and subcortical level.

As demonstrated here, an adaptive threshold provides advantages in information representation, since the variance of the response time of a neuron with an adaptive threshold is more restricted than that of a neuron with a fixed threshold, both across stimuli and across subthreshold initial states. In combination with the use of a population-based time reference, this attributes a role in generating robust information transmission to the use of an adaptive threshold.

Conversely, due to its adaptation to state, the adaptive threshold model may have less access to the state itself, which is e.g. valuable if the state value should play a role in the processing of information. This trade-off between fixed and adaptive encoding has been observed before in multiple contexts (e.g. [38,39]).

### Assumptions in information-theoretic decoding

Information theoretic methods are useful to evaluate and compare different decoding mechanisms, since they are quite general, make only few assumptions and lead to objective performance estimates. Results from a decoding method are, however, only relevant, if they are compatible with the processing performed in subsequent stations in the brain. Below we address some some limitations/caveats stemming from information theory in general and relating to the present study.

First, we used the entire spike train (binned at several temporal precisions ranging from 0.5–30 ms) as the response of the neuron. While this approach guarantees that we used all the available information, it also assumes that the decoder can wait for the entire time-span to decide whether and how to respond. A closer approximation to the decoding performance of a (point) neuron would be a decoder with a limited integration-time and a memory term, that weights recent inputs stronger than past inputs. As the processing is performed online and in real-time, multiple decoders of this kind could be lined up in sequence to perform classification of longer responses. Alternatively, one could assume that (internal) decoding only happens on short timescales and thus restricts the view to a single decoder. This would result in information loss, but it will not significantly affect the computational power of the adaptive threshold over the fixed threshold as described herein.

Second, the present comparison between adaptive and fixed threshold neurons with respect to decoding across states assumed that the responses from all states were available to the decoder. While this assumption is reasonable to assume over long time-scales, one could postulate that a decoder is instead only matched to one state (e.g. the up state), in which case robustness of the decoding strategy could then be defined as how well the state-specific representation can transfer to other states. Concretely, the decoder would specifically decode (i.e. be trained) for one state, and loss in decoding quality in another state would depend mostly on the similarity of the response between states. In this case we would predict a neuron with adaptive threshold to still encode more consistently across states, since a state change would lead to less changes in neural response.

Generally, the robustness in decoding across states could be achieved by either an adaptive threshold mechanism which leads to less changes in neural responses to state variations (as shown in current study), or by using the trial-by-trial state knowledge to discount part of the neural response variability caused by state changes (see [20]). Safaari *et al*. [20] modeled the effect of state variation on neural response explicitly, thus their approach gives a lower bound on the information gain by knowing the states; here by using mutual information analysis we gave the upper bound of the information gain the state knowledge could contribute.

### Consequences for intrinsic network dynamics

The integration and transfer characteristics of individual neurons are relevant as they form the basis of information processing on the network level. The adaptive threshold neuron's sensitivity to correlated inputs could contribute to a much discussed property of neural representation, namely sparseness. Limiting the neuron’s response to correlated input reduces its responsiveness, so it responds only to a smaller subset of input patterns/rates, thereby reducing the overall number of spikes in the network. Hence, a side-effect could be a more energy efficient operation based on the limited number of spikes.

The adaptive threshold neuron's general ability to follow changes in preceding voltage reduces the variability in the spike latency after stimulus onset as compared with a neuron with a fixed threshold. Consequently, when the same stimulus is presented, the response variability with respect to state differences is reduced (see Fig 5A). Hence, in a network with multiple layers, the dispersion of spike timing would accumulate at a lower rate (per neuron). To make use of this precision, as discussed above, the time-frame for decoding should not be fixed to the presynaptic response onset, but rather be defined by the average timing on the postsynaptic side [25].

While certain consequences of single neuron properties to the network level can be predicted well, explicit simulations or theoretical explorations are required to test these hypotheses in further detail.

### Alternate models of adaptive threshold

In the present study, an adaptive spike threshold was modeled on the basis of a phenomenological description by Fontaine et al. [2], which was shown to make accurate spike-time predictions for the case of subcortical neurons in the inferior colliculus of the Barn owl. Alternative descriptions of an adaptive threshold could be based on biophysical mechanisms, such as the inactivation of Na^+^ channels [14] and the activation of K^+^ channels (e.g. low-voltage activated Kv1 channels [15]). These descriptions would be directly based on a biological mechanism and could therefore be tested more directly using specific tools, for instance. Na+-channel blockers together with a dynamic clamp-based electrical insertion of voltage-dependent conductances [40]. However, for the present purpose, i.e. a neural decoding analysis, the phenomenological properties of the model are more relevant than its biophysical basis. As long as the Fontaine model is consistent on the phenomenological level, the present results should transfer accurately to more biologically realistic models. We used the present model because of the usual advantages of using simplified models: simplified simulation and a more direct link between an underlying mechanism and its computational consequence. In the opposite direction, despite the elegance of the Fontaine model, one may ask whether computationally simpler models could account for the adaptive threshold, which would be advantageous for large scale simulations.

### Future directions

While the present work addresses many questions relevant to the computational effects of an adaptive threshold, a range of questions remains open. In particular, including other neural constraints (e.g. limited memory) would be of interest, as well as the explicit study of network activity, and the consequences on sparseness and efficiency on the network level. Experimentally, it would be of interest to map threshold adaptiveness across different systems with a focus on directly modulating the adaptive nature, and discovering neural systems which exhibit a fixed threshold (if they exist).

## Materials and Methods

### Neural recordings

Mice from either sex were used according to the Guidelines of National Institutes of Health. Experiments were approved by the Institutional Animal Care and Use Committee.

*In vitro* whole-cell current-clamp recordings were performed in acutely prepared slices of the barrel cortex after maturation of evoked neurotransmission [41] as described before [42–44] with minor modifications. Animals were anesthetized using Isoflurane before they were decapitated. Oblique thalamocortical slices [45](300 mm) were cut 45° from the midsagittal plane in chilled low-calcium, low-sodium Ringer’s solution (in mM; sucrose, 250; KCl, 2.5; MgSO_4_⋅7H_2_O, 4; NaH_2_PO_4_⋅H_2_O, 1; HEPES, 15; D-(+)-glucose, 11; CaCl_2_, 0.1). Slices were incubated at 37°C for 45 minutes and kept in room temperature in carbonated (5% CO2 and 95% O2) bath solution (pH 7.4, normal Ringer’s solution: in mM, NaCl, 119; KCl, 2.5; MgSO_4_, 1.3; NaH_2_PO_4_, 1; NaHCO_3_, 26.3; D-(+)-glucose, 11; CaCl_2_, 2.5).

Visualized whole-cell recordings were performed using an Axoclamp-2B amplifier under an IR-DIC objective (Olympus) in room temperature. A bipolar extracellular stimulation electrode was placed in the lower half of a L4 barrel representing a mystacial vibrissa. A recording electrode (3–4 MOhm) containing an internal solution (pH 7.25; in mM; potassium gluconate, 116; KCl, 6; NaCl, 2; HEPES, 20 mM; EGTA, 0.5; MgATP, 4; NaGTP, 0.3) was placed orthogonal to the stimulation electrode within 150–300 μm of the cortical surface. For whole cell recordings, putative excitatory cells were selected based on pyramidal shaped somata, apical dendrites and distal tuft orientation, and regular pattern of spiking to somatic current injections (500 ms). The serial resistance *R*_*s*_ was compensated by bridge balance. Data was filtered (2 kHz), digitized at 5 kHz using a 12 bit National Instruments data acquisition board and acquired using Strathclyde Electrophysiology Suite for offline data analysis.

All analyses were performed offline in MATLAB (MathWorks, Inc). Raw voltage traces were smoothed using running window averaging (1 ms window size), and resting membrane potential (Vm, in mV) was calculated as the average membrane potential in a 40 ms time window prior to the stimulus onset. For those sweeps in which spike was observed, spike threshold (Vt) and spike latency (St) in respect to stimulus onset were calculated. Spike threshold was defined as the membrane potential value at which second derivative of membrane potential reached maximum as described before [7]. When analyzing neuronal responses across different resting membrane states, the data was grouped into either 3 states with 10 mV interval (-85:10:-55 mV, Fig 7A and 7B) or 5 states with 5 mV interval (-82.5:5:-57.5 mV, Fig 7E and 7G) based on the *V*_*m*_.

### Neural simulations

#### Single neuron dynamics

Unless specified, we used the exponential integrate and fire model [46] to simulate postsynaptic neuron membrane dynamics:

$$C_{m}\cdot dV_{m}/dt=-g_{L}(V_{m}-E_{L})+g_{L}\cdot \Delta_{T}exp((V_{m}-\theta )/\Delta_{T})+I$$ (1)

where *C*_*m*_ = 50 pF is the membrane capacitance, *g*_*L*_ = 10 ns is the leak conductance, *E*_*L*_ = -70 mV is the leak reversal potential, and *Δ*_*T*_ = 1 mV is the slope factor which characterize the sharpness of spike initiation. With these parameters the membrane time constant *τ*_*m*_ = 5ms. *V*_*m*_ is the membrane potential in mV, and *θ* is the spike threshold. For neurons with adaptive threshold, *θ* is determined by [2]:

$$\tau_{\theta }\cdot d\theta /dt=\theta_{\infty }(V_{m})-\theta$$ (2)

where *τ*_*θ*_ = 6 ms is the time constant of the threshold dynamics, *θ*_∞_ is the steady-state spike threshold and is determined by *V*_*m*_:

$$\theta_{\infty }(V_{m})=\alpha (V_{m}-V_{i})+V_{T}+k_{a}\cdot log(1+exp((V_{m}-V_{i})/k_{i}))$$ (3)

where α = 0.3, β = 1.1, *k*_*a*_ = 7, *k*_*i*_ = 8.75, *V*_*T*_ = -50 mV and *V*_*i*_ = -55mV were taken from Fontaine et al. [2]. For neurons with fixed spike threshold, the *θ* was set to values ranging from -52—53.8 mV, so that the overall average firing rate were comparable between adaptive and fixed spike threshold model across all conditions in one experiment. Spikes were detected when *V*_*m*_ > *θ*+3mV, and following a 0.5ms refractory period the *V*_*m*_ was reset to -70 mV. I is the input current neurons receive. All simulations were performed using 1st order Euler method with 0.1 ms time steps in MATLAB.

The Hodgkin-Huxley (HH) model was simulated using model parameters from a previously published point-conductance model [47]:

$$C_{m}∙\frac{dV_{m}}{dt}=-g_{Na}m^{3}h(V_{m}-E_{Na})-g_{K}n^{4}(V_{m}-E_{K})-g_{L}(V_{m}-E_{L})+I$$ (4)

where *C*_*m*_ = 1 μF/cm^2^ is the membrane capacitance, *g*_*Na*_ = 60 mS/cm^2^ is the maximum sodium conductance, *m* and *h* are the sodium activation and inactivation variable, respectively, and *E*_*Na*_ = 50 mV is the sodium equilibrium potential. Similarly, *g*_*K*_ = 10 mS/cm^2^ is the maximum conductance of the delayed-rectifier potassium channels, *n* is the potassium channel activation variable and *E*_*K*_ = -90 mV is the potassium equilibrium potential; *g*_*L*_ is the leak conductance and *E*_*L*_ = -70 mV is the leak equilibrium potential. We did not include the non-inactivating potassium current *I*_*M*_, since its main role is spike frequency adaptation, and we are interested in the first spikes after stimulus onset (each stimulation evoked mostly 1 spike in the majority of the trials with HH model). Including *I*_*M*_ did not change the results qualitatively. We modified the leak conductance to 0.2 mS/cm^2^ so that the effective passive membrane time constant *τ*_*m*_ = 5 ms, the same as the exponential integrate and fire model used. The dynamics of *m*, *h* and n are described by:

$$dm/dt=\alpha_{m}(V)(1-m)-\beta_{m}(V)m$$

$$dh/dt=\alpha_{h}(V)(1-h)-\beta_{h}(V)h$$

$$dn/dt=\alpha_{n}(V)(1-n)-\beta_{n}(V)n$$

where

$$\alpha_{m}(V)=-0.32(V+45)/(exp((V+45)/4)-1)$$

$$\beta_{m}(V)=0.28(V+18)/(exp((V+18)/5)-1)$$

$$\alpha_{h}(V)=0.128exp(-(V+51+a)/18)$$

$$\beta_{h}(V)=4/(1+exp(-(V+28+a)/5)$$

$$\alpha_{h}(V)=-0.032(V+43)/(exp(-(V+43/5)-1)$$

$$\beta_{h}(V)=0.5exp(-(V+48)/40).$$

The half-activation voltage *V*_*e*_, slope factor *k*_*a*_ and half-inactivation voltage *V*_*i*_, slope factor *k*_*i*_ of the Na current are obtained by fitting a Boltzmann function to the Na activation and inactivation curve in voltage range of -50~-36 mV [17], respectively. The value of *V*_*i*_ was modified by changing the value of *a*; in the original model *a* = 0, and calculated *V*_*i*_ = -46 mV. With these model parameters, the equivalent *k*_*a*_ = 3.2, *k*_*i*_ = 4.1, and *V*_*T*_ = -55 mV (see [17]). The simulations with HH model were performed using 1st order Euler method with 0.01 ms time steps in MATLAB.

#### Network structure

To study the properties of neurons with or without adaptive threshold, we constructed a simple feed-forward excitatory neural network, where 100 presynaptic input neurons connect to a single postsynaptic neuron with one synaptic connection each. All connections were identical (amplitude, 14 pA; failure rate, 0.03; coefficient of variation of amplitude, 0.3). Connection probabilities and strength resemble typical layer 4—layer 2/3 excitatory feed-forward connections found in rat barrel cortex [48] except for the synaptic latencies, which were normally distributed with a standard deviation σ ranged from 0–4 ms. When presynaptic neurons fire at the same time, the normally distributed synaptic delays effectively result in excitatory postsynaptic currents (EPSCs) arriving at postsynaptic neuron with a temporal jitter of σ. The EPSC was modeled by an exponential function with time constant of 5 ms, and we did not include short-term synaptic dynamics in the model.

#### Stimulation and network activity

We considered two types of presynaptic encoding strategies [49,50]: 1) a classic “rate encoding”, in which the stimulus was defined as number of presynaptic neurons activated in each trial, and across trials a random subset of presynaptic neurons were selected to be active. In this case the postsynaptic neuron tries to decode how many presynaptic neurons are active in any given trial. 2) a “pattern encoding” (or “temporal encoding”), in which the stimulus was defined as presynaptic activation pattern, i.e. in response to the same stimulus the same neuron had exactly the same activity across trials, and numbers of activated presynaptic neurons were the same across different stimuli. Under this condition the postsynaptic cell tried to decode which presynaptic activity pattern was present in any given trial. Under both conditions, when a presynaptic neuron was decided to be activated in a given trial it generated a spike at a fixed time, which was 60 ms after stimulation started. In other word, the temporal delay of action potential arrival time at postsynaptic neuron was purely caused by the synaptic latency; the presynaptic spike time was chosen so that the postsynaptic neuron was at steady state when presynaptic cells spiked. The activation state of the postsynaptic neuron was modulated by setting the leak reversal potential *E*_*L*_ to different values; mathematically it is equivalent to drive the cell with a constant current. Each stimulus was delivered 150 times for a given condition (different σ and/or different states); for each condition we simulated 500 such randomly generated networks.

Noise in the network was introduced by inserting spikes generated from a Poisson process in the presynaptic neurons. The mean event rate of the Poisson process (expressed as mean noise spike rate from a single presynaptic neuron in Hz) determined the overall noise strength.

### Analysis of stimulus capacity/ mutual information analysis

We employed the Shannon information theory [51] to analyze the stimulus representation capacity of neurons with either adaptive or fixed spike threshold. The mutual information *MI* between the stimulus *S* and neuronal response *R* is calculated as

I(S,R)=H(R)−H(R|S)

in which *H(R)* denotes the entropy of the response variable *R*:

$$H(R)=-\sum_{i=1}^{n}p(r_{i}){log}_{2}(p(r_{i}))$$

and *H(R|S)* is given as

$$H(R|S)=-\sum_{i=1}^{n}p(s_{i})\sum_{j=1}^{m}p(r_{j}|s_{i}){log}_{2}(p(r_{j}|s_{i}))$$

where *m*, *n* is the number of possible response and stimulus patterns and *p(r)* and *p(s)* is the occurrence probability of these patterns, respectively. Intuitively, the mutual information measures how much uncertainty about one variable, either *R* or *S*, can be reduced by an ideal observer observing the other variable. It is theoretically the upper bound of knowledge can be gained from the neuronal response under a given condition (i.e. no other source of additional information).

To construct response patterns, we binned the neuronal response spike train from individual trials using various temporal bin sizes (1 ms, 2 ms, 5 ms or 30 ms). The analysis window was 0–30 ms after stimulus onset, which included all spikes elicited by the stimulus. Number of spikes in each temporal bin was counted, and the resulting numeric vector with different length, depending on temporal bin size, was used to calculate the mutual information. When calculating the mutual information between stimulus and neural response knowing the states (Figs 5–7), the states was supplied to the binned response vector as an additional dimension. The mutual information calculation was performed using Spike Train Analysis toolbox [52] with shuffle correction combined with Panzeri-Treves estimator [53] as bias correction method [54].

With the bias correction method employed, the number of trials per stimulus (*N*_*s*_) should be at least ¼ of the number of total possible response patterns (*N*_*R*_) to obtain an accurate estimation of the mutual information [54]. In our simulations a neuron fires at most 2 spikes in each trial. Using a 30 ms analysis window and 2 ms bin size, the *N*_*R*_ would be $(\frac{15}{2})+(\frac{15}{1})+(\frac{15}{0})=121$, thus the minimum *N*_*s*_ required is 31. If a bin size of 1 ms is used, then *N*_*R*_ = 466 and the minimum *N*_*s*_ would be 117. With *N*_*s*_ = 150 in our dataset, the calculated mutual information should be bias-free. We ran a set of simulations with *N*_*s*_ = 1000 and compared the *MI* calculated using *N*_*s*_ = 1000 and those calculated with various smaller *N*_*s*_ using bootstrapping method, setting analysis window to 30 ms and bin size to 1 ms. At *N*_*s*_ = 150, the difference is already <0.1% (<4x10^-3^ bit in absolute value) of the true *MI*.

The total information that can be decoded without knowing the state is given by *I(S; R)*, which is best interpreted as the information robust to state changes, i.e. the information can be decoded without paying attention to state. The total information that can be decoded knowing the state in addition, is given by *I(S; R*, *states)*. We calculated the robustness index *RI*, which is defined as I(S; R)/I(S; R, states). *RI* values close to 1 imply a high robustness of the decodable information to not knowing states, i.e. most of the total information can be obtained without the state knowledge. Conversely, low *RI* values indicate that taking state into account significantly adds to the amount of information the postsynaptic neuron transmits. Since RI is relative to I(S; R, states) and both constituents are positive, RI is bounded in [0,1].

The decoding with moving reference time was implemented by taking the average spike time of all simulated neurons (N = 500) in response to the stimulus. Assuming a flat distribution of tuning preferences, this average time will even be stimulus independent. The early part of the decoding window (set of discretization bins) was then aligned to the average response time (similar to [25]). Note, that the neural responses could differ by state and thus also shift the average response time forward or backward.

## Supporting Information

S1 Fig Hodgkin-Huxley model can exhibit adaptive or fixed behavior, depending on the parameter range. We varied the inactivation dynamics of the sodium conductance via its half-inactivation voltage V_i_, selecting a relevant set of values in the range between -58 and -46mV. The relationship between V_i_ and V_T_ (approximate spike threshold inferred from model parameters, see Platkiewicz and Brette, 2010) determines the type of threshold behavior exhibited. If V_i_ < V_T_ the threshold is more adaptive, if V_i_ > V_T_, the threshold is close to fixed (here V_T_ = -55mV, Platkiewicz and Brette, 2010). **(A)** In the rate encoding case, the information represented in the neural response shows a stronger low-pass behavior for the adaptive (red, V_i_ = -58mV) parameters setting, compared with the fixed (blue, V_i_ = -46mV), corresponding well to the relation between the adaptive and fixed threshold model in the main text (compare to Fig 3A). **(B)** The transition of the edge of MI to wider σ occurs gradually as a function of V_i_. **(C)** In the pattern encoding case, MI shows a bandpass behavior, again quite similar to the models in the main text. The adaptive parameter setting (red) has a preference for lower σ, compared to the fixed threshold parameters setting (blue). **(D)** Increasing V_i_ from the adaptive (-58mV) to the fixed threshold (-46 mV) region gradually increases the σ for maximal MI.
(TIFF)

S2 Fig Size of input population affects pattern and rate encoding differently, but not adaptive and fixed threshold neurons. The number of neurons contributing to a neural input will influence the temporal dispersion of the EPSP. Large numbers will approximate their governing distribution (here a Gaussian distribution) well. **(A)** For a rate encoding, larger input populations therefore lead to an improved information content, since trial-to-trial variation (here: different selections of contributing neurons) is overcome by the average input (dark colors). For small populations, the low number of inputs together with their temporal dispersion lead to low information content (bright colors) The ranges in the legend indicate the number of active neurons for each stimulus. Each range was divided into 6 equally spaced steps to form the different stimuli, e.g. 20–30 indicates the use of [20,22,24,26,28,30] active neurons to define the 6 stimuli differing in rate. In order to keep the total input the same, the single EPSP was correspondingly reduced (see right axis in plots B2/D2). **(B)** Population size has a similar effect on the adaptive and fixed threshold model (B1, B2), as indicated also by the invariant shape of their difference (B3). **(C)** For the pattern encoding, the population size has an inverse effect on represented information: Small input populations lead to large MI (bright colors), while large populations lead to small MI (dark colors). For a large population, different patterns approximate the governing distribution well, and are thus hard to distinguish on their combined EPSC. On the other hand, small populations, produce distinguishable patterns of spikes in the present setting, since spike times are fixed across trials. **(D)** Similar to the rate encoding case, the size of the input population did not affect the adaptive and fixed threshold neurons differentially.
(TIFF)

## References

[1] Destexhe A, Rudolph M, Paré D. The high-conductance state of neocortical neurons in vivo. Nat Rev Neurosci. 2003;4: 739–751. 12951566 10.1038/nrn1198

[2] Fontaine B, Peña JL, Brette R. Spike-threshold adaptation predicted by membrane potential dynamics in vivo. PLoS Comput Biol. 2014;10: e1003560 10.1371/journal.pcbi.1003560 24722397 PMC3983065

[3] Spruston N. Pyramidal neurons: dendritic structure and synaptic integration. Nat Rev Neurosci. 2008;9: 206–221. 10.1038/nrn2286 18270515

[4] Goldberg EM, Clark BD, Zagha E, Nahmani M, Erisir A, Rudy B. K+ channels at the axon initial segment dampen near-threshold excitability of neocortical fast-spiking GABAergic interneurons. Neuron. 2008;58: 387–400. 10.1016/j.neuron.2008.03.003 18466749 PMC2730466

[5] Azouz R, Gray CM. Dynamic spike threshold reveals a mechanism for synaptic coincidence detection in cortical neurons in vivo. Proc Natl Acad Sci U S A. 2000;97: 8110–8115. 10859358 10.1073/pnas.130200797 PMC16678

[6] Azouz R, Gray CM. Adaptive Coincidence Detection and Dynamic Gain Control in Visual Cortical Neurons In Vivo. Neuron. 2003;37: 513–523. 12575957 10.1016/s0896-6273(02)01186-8

[7] Wilent WB, Contreras D. Stimulus-dependent changes in spike threshold enhance feature selectivity in rat barrel cortex neurons. J Neurosci. 2005;25: 2983–2991. 15772358 10.1523/JNEUROSCI.4906-04.2005 PMC6725135

[8] de Polavieja GG, Harsch A, Kleppe I, Robinson HPC, Juusola M. Stimulus history reliably shapes action potential waveforms of cortical neurons. J Neurosci. 2005;25: 5657–5665. 15944394 10.1523/JNEUROSCI.0242-05.2005 PMC6724966

[9] Henze DA, Buzsáki G. Action potential threshold of hippocampal pyramidal cells in vivo is increased by recent spiking activity. Neuroscience. 2001;105: 121–130. 11483306 10.1016/s0306-4522(01)00167-1

[10] Farries MA, Kita H, Wilson CJ. Dynamic spike threshold and zero membrane slope conductance shape the response of subthalamic neurons to cortical input. J Neurosci. 2010;30: 13180–13191. 10.1523/JNEUROSCI.1909-10.2010 20881137 PMC2966473

[11] Brenner N, Bialek W, de Ruyter van Steveninck R. Adaptive Rescaling Maximizes Information Transmission. Neuron. 2000;26: 695–702. 10896164 10.1016/s0896-6273(00)81205-2

[12] Benda J, Maler L, Longtin A. Linear versus nonlinear signal transmission in neuron models with adaptation currents or dynamic thresholds. J Neurophysiol. 2010;104: 2806–2820. 10.1152/jn.00240.2010 21045213

[13] Bohte SM. Efficient spike-coding with multiplicative adaptation in a spike response model. In Advances in Neural Information Processing Systems 2012; 25: 1835–1843.

[14] Platkiewicz J, Brette R. Impact of fast sodium channel inactivation on spike threshold dynamics and synaptic integration. PLoS Comput Biol. 2011;7: e1001129 10.1371/journal.pcbi.1001129 21573200 PMC3088652

[15] Higgs MH, Spain WJ. Kv1 channels control spike threshold dynamics and spike timing in cortical pyramidal neurones. J Physiol. 2011;589: 5125–5142. 10.1113/jphysiol.2011.216721 21911608 PMC3225669

[16] Fontaine B, MacLeod KM, Lubejko ST, Steinberg LJ, Köppl C, Peña JL. Emergence of band-pass filtering through adaptive spiking in the owl’s cochlear nucleus. J Neurophysiol. 2014;112: 430–445. 10.1152/jn.00132.2014 24790170 PMC4064407

[17] Platkiewicz J, Jonathan P, Romain B. A Threshold Equation for Action Potential Initiation. PLoS Comput Biol. 2010;6: e1000850 10.1371/journal.pcbi.1000850 20628619 PMC2900290

[18] Hasenstaub A, Sachdev RNS, McCormick DA. State changes rapidly modulate cortical neuronal responsiveness. J Neurosci. 2007;27: 9607–9622. 17804621 10.1523/JNEUROSCI.2184-07.2007 PMC6672966

[19] Tan AYY, Chen Y, Scholl B, Seidemann E, Priebe NJ. Sensory stimulation shifts visual cortex from synchronous to asynchronous states. Nature. 2014;509: 226–229. 10.1038/nature13159 24695217 PMC4067243

[20] Safaai H, Neves R, Eschenko O, Logothetis NK, Panzeri S. Modeling the effect of locus coeruleus firing on cortical state dynamics and single-trial sensory processing. Proc Natl Acad Sci U S A. 2015;112: 12834–12839. 10.1073/pnas.1516539112 26417078 PMC4611622

[21] Panzeri S, Petersen RS, Schultz SR, Lebedev M, Diamond ME. The role of spike timing in the coding of stimulus location in rat somatosensory cortex. Neuron. 2001;29: 769–777. 11301035 10.1016/s0896-6273(01)00251-3

[22] Petersen RS, Panzeri S, Diamond ME. Population Coding of Stimulus Location in Rat Somatosensory Cortex. Neuron. 2001;32: 503–514. 11709160 10.1016/s0896-6273(01)00481-0

[23] Quian Quiroga R, Panzeri S. Extracting information from neuronal populations: information theory and decoding approaches. Nat Rev Neurosci. 2009;10: 173–185. 10.1038/nrn2578 19229240

[24] Thorpe S, Delorme A, Van Rullen R. Spike-based strategies for rapid processing. Neural Netw. 2001;14: 715–725. 11665765 10.1016/s0893-6080(01)00083-1

[25] Panzeri S, Diamond ME. Information Carried by Population Spike Times in the Whisker Sensory Cortex can be Decoded Without Knowledge of Stimulus Time. Front Synaptic Neurosci. 2010;2: 17 10.3389/fnsyn.2010.00017 21423503 PMC3059688

[26] Hu W, Tian C, Li T, Yang M, Hou H, Shu Y. Distinct contributions of Na(v)1.6 and Na(v)1.2 in action potential initiation and backpropagation. Nat Neurosci. 2009;12: 996–1002. 10.1038/nn.2359 19633666

[27] McGinley MJ, Vinck M, Reimer J, Batista-Brito R, Zagha E, Cadwell CR, et al Waking State: Rapid Variations Modulate Neural and Behavioral Responses. Neuron. 2015;87: 1143–1161. 10.1016/j.neuron.2015.09.012 26402600 PMC4718218

[28] Yamashita T, Pala A, Pedrido L, Kremer Y, Welker E, Petersen CCH. Membrane potential dynamics of neocortical projection neurons driving target-specific signals. Neuron. 2013;80: 1477–1490. 10.1016/j.neuron.2013.10.059 24360548

[29] Robinson RB, Siegelbaum SA. Hyperpolarization-activated cation currents: from molecules to physiological function. Annu Rev Physiol. 2003;65: 453–480. 12471170 10.1146/annurev.physiol.65.092101.142734

[30] Levitan H, Segundo JP, Moore GP, Perkel DH. Statistical analysis of membrane potential fluctuations. Relation with presynaptic spike train. Biophys J. 1968;8: 1256–1274. 4301347 10.1016/S0006-3495(68)86554-3 PMC1367693

[31] Svirskis G, Rinzel J. Influence of temporal correlation of synaptic input on the rate and variability of firing in neurons. Biophys J. 2000;79: 629–637. 10919997 10.1016/S0006-3495(00)76321-1 PMC1300963

[32] Voigts J, Sakmann B, Celikel T. Unsupervised whisker tracking in unrestrained behaving animals. J Neurophysiol. 2008;100: 504–515. 10.1152/jn.00012.2008 18463190

[33] Voigts J, Herman DH, Celikel T. Tactile object localization by anticipatory whisker motion. J Neurophysiol. 2015;113: 620–632. 10.1152/jn.00241.2014 25339711

[34] Celikel T, Sakmann B. Sensory integration across space and in time for decision making in the somatosensory system of rodents. Proc Natl Acad Sci U S A. 2007;104: 1395–1400. 17227858 10.1073/pnas.0610267104 PMC1783091

[35] Engel AK, König P, Kreiter AK, Singer W. Interhemispheric synchronization of oscillatory neuronal responses in cat visual cortex. Science. 1991;252: 1177–1179. 2031188 10.1126/science.252.5009.1177

[36] Lakatos P, Chen C-M, O’Connell MN, Mills A, Schroeder CE. Neuronal oscillations and multisensory interaction in primary auditory cortex. Neuron. 2007;53: 279–292. 17224408 10.1016/j.neuron.2006.12.011 PMC3717319

[37] Lakatos P, Karmos G, Mehta AD, Ulbert I, Schroeder CE. Entrainment of neuronal oscillations as a mechanism of attentional selection. Science. 2008;320: 110–113. 10.1126/science.1154735 18388295

[38] Fairhall AL, Lewen GD, Bialek W, de Ruyter Van Steveninck RR. Efficiency and ambiguity in an adaptive neural code. Nature. 2001;412: 787–792. 11518957 10.1038/35090500

[39] Hildebrandt KJ, Ronacher B, Hennig RM, Benda J. A neural mechanism for time-window separation resolves ambiguity of adaptive coding. PLoS Biol. 2015;13: e1002096 10.1371/journal.pbio.1002096 25761097 PMC4356587

[40] Sharp AA, O’Neil MB, Abbott LF, Marder E. Dynamic clamp: computer-generated conductances in real neurons. J Neurophysiol. 1993;69: 992–995. 8463821 10.1152/jn.1993.69.3.992

[41] Martens MB, Celikel T, Tiesinga PHE. A Developmental Switch for Hebbian Plasticity. PLoS Comput Biol. 2015;11: e1004386 10.1371/journal.pcbi.1004386 26172394 PMC4501799

[42] Allen CB, Celikel T, Feldman DE. Long-term depression induced by sensory deprivation during cortical map plasticity in vivo. Nat Neurosci. 2003;6: 291–299. 12577061 10.1038/nn1012

[43] Celikel T, Szostak VA, Feldman DE. Modulation of spike timing by sensory deprivation during induction of cortical map plasticity. Nat Neurosci. 2004;7: 534–541. 15064767 10.1038/nn1222 PMC3082358

[44] Clem RL, Celikel T, Barth AL. Ongoing in vivo experience triggers synaptic metaplasticity in the neocortex. Science. 2008;319: 101–104. 10.1126/science.1143808 18174444

[45] Finnerty GT, Roberts LS, Connors BW. Sensory experience modifies the short-term dynamics of neocortical synapses. Nature. 1999;400: 367–371. 10432115 10.1038/22553

[46] Fourcaud-Trocmé N, Hansel D, van Vreeswijk C, Brunel N. How spike generation mechanisms determine the neuronal response to fluctuating inputs. J Neurosci. 2003;23: 11628–11640. 14684865 10.1523/JNEUROSCI.23-37-11628.2003 PMC6740955

[47] Destexhe A, Paré D. Impact of network activity on the integrative properties of neocortical pyramidal neurons in vivo. J Neurophysiol. 1999;81: 1531–1547. 10200189 10.1152/jn.1999.81.4.1531

[48] Feldmeyer D, Lübke J, Silver RA, Sakmann B. Synaptic connections between layer 4 spiny neurone- layer 2/3 pyramidal cell pairs in juvenile rat barrel cortex: physiology and anatomy of interlaminar signalling within a cortical column. J Physiol. 2002;538: 803–822. 11826166 10.1113/jphysiol.2001.012959 PMC2290091

[49] Theunissen F, Miller JP. Temporal encoding in nervous systems: a rigorous definition. J Comput Neurosci. 1995;2: 149–162. 8521284 10.1007/BF00961885

[50] Panzeri S, Brunel N, Logothetis NK, Kayser C. Sensory neural codes using multiplexed temporal scales. Trends Neurosci. 2010;33: 111–120. 10.1016/j.tins.2009.12.001 20045201

[51] Shannon CE. A mathematical theory of communication. The Bell System Technical Journal 1948; 27(1): 379–423.

[52] Goldberg DH, Victor JD, Gardner EP, Gardner D. Spike train analysis toolkit: enabling wider application of information-theoretic techniques to neurophysiology. Neuroinformatics. 2009;7: 165–178. 10.1007/s12021-009-9049-y 19475519 PMC2818590

[53] Panzeri S, Treves A. Analytical estimates of limited sampling biases i different information measures. Network: Comput. Neural Syst. 1996; 7: 87–107. 10.1080/0954898X.1996.11978656 29480146

[54] Ince RAA, Senatore R, Arabzadeh E, Montani F, Diamond ME, Panzeri S. Information-theoretic methods for studying population codes. Neural Netw. 2010;23: 713–727. 10.1016/j.neunet.2010.05.008 20542408

