# Supplementary figures and images for: Adaptive Spike Threshold Enables Robust and Temporally Precise Neuronal Encoding
Source: PLoS Comput Biol. 2016 Jun 15;12(6):e1004984. doi: 10.1371/journal.pcbi.1004984 (PMC4909286; doi:10.1371/journal.pcbi.1004984)

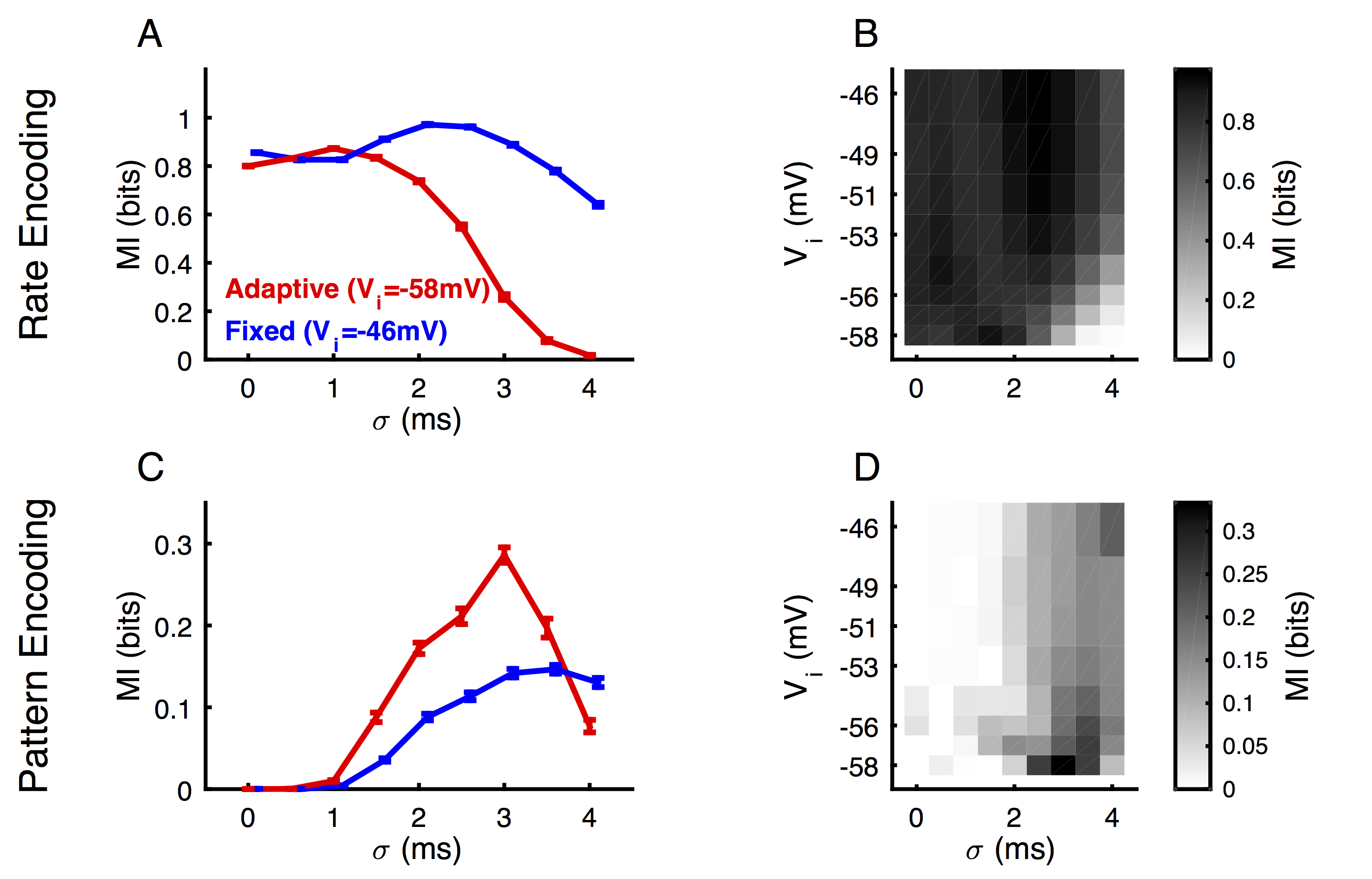

Supplement: S1 Fig — We varied the inactivation dynamics of the sodium conductance via its half-inactivation voltage Vi, selecting a relevant set of values in the range between -58 and -46mV. The relationship between Vi and VT (approximate spike threshold inferred from model parameters, see Platkiewicz and Brette, 2010) determines the type of threshold behavior exhibited. If Vi < VT the threshold is more adaptive, if Vi > VT, the threshold is close to fixed (here VT = -55mV, Platkiewicz and Brette, 2010). (A) In the rate encoding case, the information represented in the neural response shows a stronger low-pass behavior for the adaptive (red, Vi = -58mV) parameters setting, compared with the fixed (blue, Vi = -46mV), corresponding well to the relation between the adaptive and fixed threshold model in the main text (compare to Fig 3A). (B) The transition of the edge of MI to wider σ occurs gradually as a function of Vi. (C) In the pattern encoding case, MI shows a bandpass behavior, again quite similar to the models in the main text. The adaptive parameter setting (red) has a preference for lower σ, compared to the fixed threshold parameters setting (blue). (D) Increasing Vi from the adaptive (-58mV) to the fixed threshold (-46 mV) region gradually increases the σ for maximal MI. (TIFF) [file pcbi.1004984.s001.tiff]

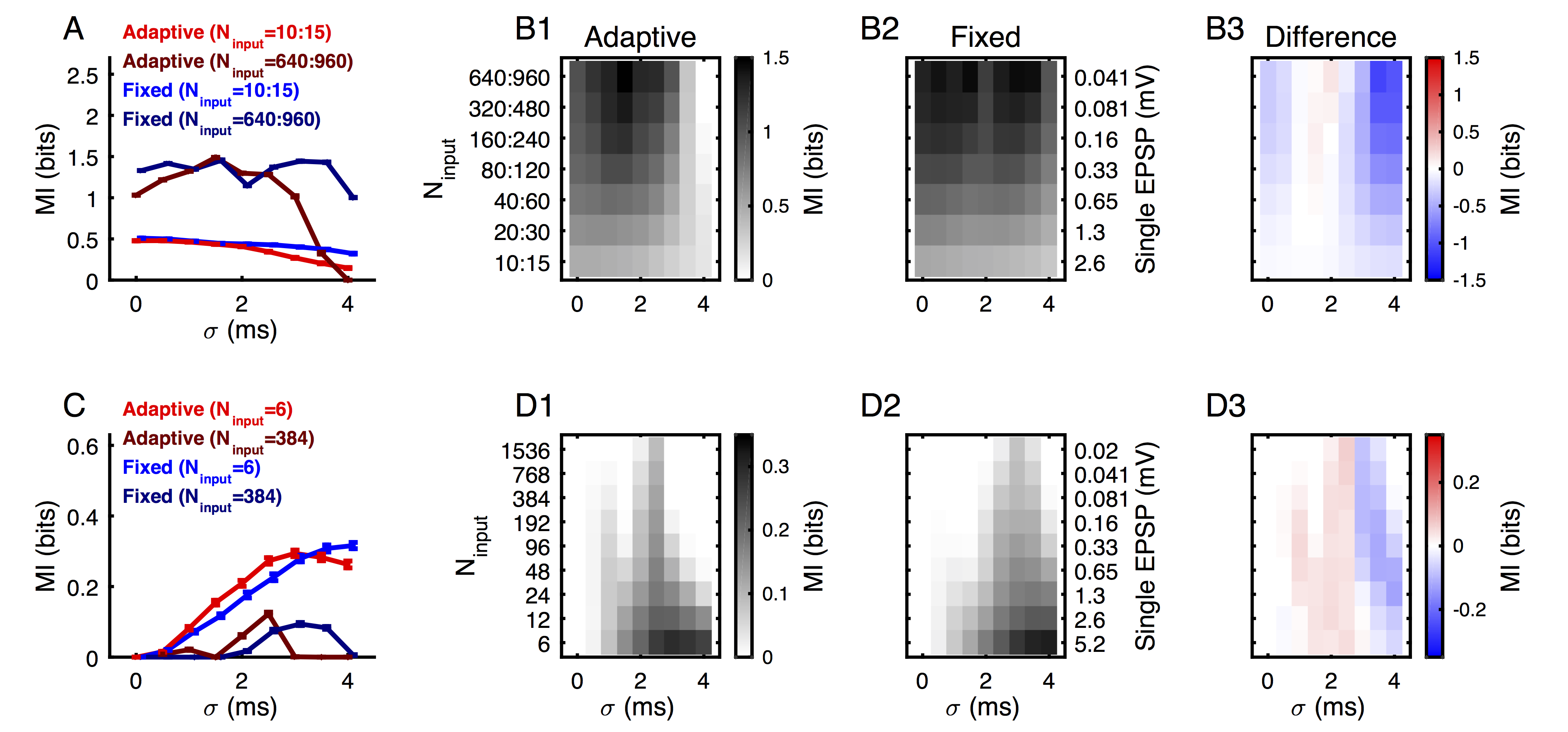

Supplement: S2 Fig — The number of neurons contributing to a neural input will influence the temporal dispersion of the EPSP. Large numbers will approximate their governing distribution (here a Gaussian distribution) well. (A) For a rate encoding, larger input populations therefore lead to an improved information content, since trial-to-trial variation (here: different selections of contributing neurons) is overcome by the average input (dark colors). For small populations, the low number of inputs together with their temporal dispersion lead to low information content (bright colors) The ranges in the legend indicate the number of active neurons for each stimulus. Each range was divided into 6 equally spaced steps to form the different stimuli, e.g. 20–30 indicates the use of [20,22,24,26,28,30] active neurons to define the 6 stimuli differing in rate. In order to keep the total input the same, the single EPSP was correspondingly reduced (see right axis in plots B2/D2). (B) Population size has a similar effect on the adaptive and fixed threshold model (B1, B2), as indicated also by the invariant shape of their difference (B3). (C) For the pattern encoding, the population size has an inverse effect on represented information: Small input populations lead to large MI (bright colors), while large populations lead to small MI (dark colors). For a large population, different patterns approximate the governing distribution well, and are thus hard to distinguish on their combined EPSC. On the other hand, small populations, produce distinguishable patterns of spikes in the present setting, since spike times are fixed across trials. (D) Similar to the rate encoding case, the size of the input population did not affect the adaptive and fixed threshold neurons differentially. (TIFF) [file pcbi.1004984.s002.tiff]
